# Effects of preventive nutrition interventions among adolescents on health and nutritional status in low‐ and middle‐income countries: A systematic review

**DOI:** 10.1002/cl2.1085

**Published:** 2020-05-18

**Authors:** Rehana A. Salam, Jai K. Das, Omar Irfan, Wardah Ahmed, Sana S. Sheikh, Zulfiqar A. Bhutta

**Affiliations:** ^1^ Division of Women and Child Health Aga Khan University Hospital Karachi Pakistan; ^2^ Department of Pediatrics Aga Khan University Karachi Pakistan; ^3^ Centre for Global Child Health The Hospital for Sick Children Toronto Canada

## Abstract

**Background:**

Malnutrition is one of the most common causes of morbidity and mortality among children and adolescents and is now considered to be one of the largest risk factors responsible for the global burden of diseases along with poor diet.

**Objectives:**

The objective of this review was to assess the impact of preventive nutrition interventions (including nutrition education and counselling; micronutrient supplementation/fortification and macronutrient supplementation) to improve the health and nutritional status of adolescents aged 10–19 years in low‐ and middle‐income countries (LMICs). The secondary objective of the review was to assess various contextual factors based on the World Health Organisation (WHO) health system building blocks framework that might potentially impact the effectiveness of these interventions for this age group.

**Search Methods:**

The search was conducted on Cochrane Controlled Trials Register (CENTRAL), MEDLINE, EMBASE, CINAHL, PsycINFO, the WHO nutrition databases, CAB Global Health, Social Science Citation Index, Scopus, WHO Global Health Index, ADOLEC and EPPI until February 5, 2019. We searched Google Scholar along with key nutrition agencies database such as Nutrition International, the Global Alliance for Improved Nutrition, the World Food Programme and HarvestPlus to search for nonindexed, grey literature to locate relevant programme evaluations and any additional trials. All searches were performed without any restrictions on publication date, language or publication status.

**Selection Criteria:**

We included randomised controlled trials, quasiexperimental studies, controlled before‐after studies and interrupted time series evaluating the effectiveness of preventive nutrition interventions among adolescents between 10 and 19 years of age from LMICs.

**Data Collection and Analysis:**

Two review authors independently assessed trials for inclusion, assessed risk of bias and extracted data from included studies. Meta‐analysis was conducted separately for each outcome and intervention. For dichotomous data, we reported risk ratios (RR) with 95% confidence intervals (CI). For continuous data, we reported the mean difference (MD) or standard mean difference (SMD) with 95% CI.

**Main Results:**

This review summarises findings from a total of 10 studies from 15 papers including 10,802 participants. All the studies included in this review assessed the impact of micronutrient supplementation/fortification on health and nutritional status among adolescents in LMIC. We did not find any study assessing the impact of nutrition education and counselling or on macronutrient supplementation among adolescents. Micronutrient supplementation/fortification interventions included calcium/vitamin D supplementation/fortification, iron supplementation with or without folic acid, zinc supplementation and multiple micronutrient (MMN) fortification. The majority of the studies (eight out of 10 studies) included adolescent girls aged between 10 and 19 years of age. We did not find any large scale preventive nutrition intervention programmes targeting adolescents in LMICs. We are uncertain of the effect of iron supplementation with or without folic acid on anaemia (daily supplementation; RR: 1.04, 95% CI 0.88, 1.24; one study; 1,160 participants; low quality evidence. Weekly supplementation; RR: 1.07, 95% CI: 0.91, 1.26; one study; 1,247 participants; low quality evidence). We are uncertain of the effect of various micronutrient supplementation/fortification on body mass index (calcium/vitamin D supplementation; (MD: −0.01 kg/m^2^; 95% CI: −1.20, 1.17; two studies; 730 participants; *I* ^2^ 94%; very low quality evidence, iron supplementation with or without folic acid; MD: 0.29 kg/m^2^; 95% CI: −0.25, 0.83; two studies; 652 participants; *I* ^2^ 69%; very low quality evidence, zinc supplementation; MD: 0.35 kg/m^2^; 95% CI: −0.15, 0.85; one study; 382 participants; very low quality evidence) and MMN fortification; MD: 0.23 kg/m^2^, 95% CI: −0.11, 0.57; two studies; 943 participants; *I* ^2^ 22%; very low quality evidence). None of the included studies reported any other primary outcomes including morbidity or adverse effects. Iron supplementation with or without folic acid may improve haemoglobin concentrations (MD: 0.42 g/dL, 95% CI: 0.13, 0.71; four studies; 1,020 participants; *I* ^2^ 89%; low quality evidence). Calcium/vitamin D supplementation may improve serum 25(OH) D levels (standardised mean difference [SMD]: 2.85, 95% CI: 0.89, 4.82; two studies; 395 participants; *I* ^2^ 99%; low quality evidence). We are uncertain of the effect of calcium only supplementation (MD: 0.02 g/cm^2^, 95% CI: −0.00, 0.04; one study; 233 participants; low quality outcome) and calcium + vitamin D supplementation (MD: 0.02 g/cm^2^, 95% CI: −0.00, 0.04; one study; 235 participants; low quality evidence) on total bone mineral density (BMD). We are uncertain of the effect of MMN fortification on haemoglobin concentrations (MD: −0.10 g/dL, 95% CI: −0.88, 0.68; two studies; 1102 participants; *I* ^2^ 100%; very low quality evidence); calcium supplementation on total body bone mineral content (BMC); (MD: 30.20 g, 95% CI: −40.56, 100.96; one study; 233 participants; low quality evidence), calcium + vitamin D supplementation on total body BMC (MD: 21.60 g, 95% CI: −45.32, 88.52; one study; 235 participants; low quality evidence) and zinc supplementation on serum zinc levels (SMD: 6.94, 95% CI: −4.84, 18.71; two studies; 494 participants; very low quality evidence). One study reported the impact of iron supplementation with or without folic acid on cognition of adolescent girls suggesting improved cognition in most of the tests with daily or twice weekly supplementation compared to once weekly or no supplementation. None of the other secondary outcomes were reported including any other development outcomes and all‐cause mortality. These findings warrant caution while interpreting due to very few studies and high heterogeneity.

**Authors' Conclusions:**

There is limited evidence of micronutrient supplementation/fortification among adolescents on health and nutritional status in LMICs, with lack of evidence on nutrition education and counselling and macronutrient supplementation. The findings are generaliseable for adolescent girls since all studies (except one) targeted female adolescents.

## 1 PLAIN LANGUAGE SUMMARY

### 1.1 Evidence is scarce on preventive nutrition interventions for adolescents in low‐ and middle‐income countries (LMICs)

Malnutrition is one of the most common causes of morbidity and mortality among adolescents in LMICs. Preventive measures include nutrition education and counselling; micronutrient supplementation/fortification and macronutrient supplementation. There are few studies assessing micronutrient supplementation and fortification programmes. What studies there are, are of low quality and generally find no effects.

There are no studies of other preventive measures, that is, macronutrient supplementation or nutrition education and counselling.

#### 1.1.1 What is this review about?

Malnutrition is one of the most common causes of morbidity and mortality among adolescents and is now considered to be one of the largest risk factors responsible for the global burden of disease, along with poor diet. This review assesses the impact of preventive nutrition interventions (including nutrition education and counselling; micronutrient supplementation/fortification and macronutrient supplementation) to improve the health and nutritional status of adolescents aged 10–19 years in LMICs.

#### 1.1.2 What is the aim of this review?

This Campbell systematic review summarises findings from 10 studies on preventive nutrition interventions among adolescents in LMICs.

#### 1.1.3 What studies are included?

To be eligible for inclusion, studies had to be randomised controlled trials (RCTs), quasiexperimental studies, controlled before‐after (CBA) studies or interrupted time series (ITS) studies evaluating the effectiveness of preventive nutrition interventions among adolescents between 10 and 19 years of age, from LMICs.

The review summarises evidence from 10 studies from 15 papers, which included 10,802 participants. All the included studies are RCTs assessing micronutrient supplementation and fortification. Adolescents girls were the intervention groups for all but one of the included studies.

No studies evaluating macronutrient supplementation or nutrition education and counselling were found.

#### 1.1.4 Do micronutrient supplementation and fortification improve health and nutritional outcomes?

Overall, the evaluated interventions mostly did not have a significant positive effect on the assessed outcomes, although this conclusion is based on a few studies of low or very low quality.

Specifically, there was no positive impact on any of the following outcomes:

Anaemia: No effect from iron supplementation with or without folic acid given daily or weekly

Body mass index (BMI): No effect from any of calcium/vitamin D, iron supplementation with or without folic acid, zinc supplementation, multiple micronutrient (MMN) fortification

Bone mineral density (BMD): No effect from any of calcium only supplementation or calcium and vitamin D supplementation. Positive effects from calcium/vitamin D supplementation were found on serum 25(OH)D level.

#### 1.1.5 What do the findings of this review mean?

The evidence on preventive nutrition interventions among adolescents from LMICs is too scarce for any conclusive implications for practice. The existing evidence is limited to micronutrient supplementation/fortification only. There is no evidence on nutrition education and counselling and macronutrient supplementation among adolescents.

Future studies assessing preventive nutrition interventions among adolescents in LMICs should focus on nutrition education and macronutrient supplementation. Future studies should be designed with longer follow‐up periods and also assess any adverse effects.

There is a need for large‐scale nutrition intervention programme evaluations from LMIC settings. Programmes targeting adolescents in LMICs should also report on contextual factors in planning, implementation and evaluation in light of the WHO health system building blocks. Future studies should target adolescent boys and girls.

#### 1.1.6 How up‐to‐date is this review?

The review authors searched for studies published up to February 2019.

## 2 BACKGROUND

### 2.1 Description of the condition

Malnutrition is one of the most common causes of morbidity and mortality among children and adolescents (UNICEF, 2005) and along with poor diet, it is now considered to be the largest risk factor responsible for the global burden of diseases (Forouzanfar Mohammad et al., 2015). A survey conducted among adolescents aged 12–15 years from 57 LMICs between 2003 and 2013 suggested that the prevalence of stunting was 10.2% while thinness was 5.5% (Caleyachetty et al., 2018). Micronutrient deficiencies account for a substantial global burden of diseases, with iron and vitamin A deficiency being among the 15 leading causes of global morbidity and mortality (WHO 2002). More than 2 billion people, including both children and adolescents, suffer from micronutrient deficiencies in the developing world (Stanger et al., 2009). In 2014, iron deficiency anaemia was one of the three most common causes of disability‐adjusted life years (DALYs) lost among adolescents along with other micronutrient deficiencies accounting for over 2,500 DALYs per 100,000 adolescents (Akseer, Al‐Gashm, Mehta, Mokdad, & Bhutta, 2017; WHO, 2014).

Adolescence is a critical age group with key changes in health and its determinants later in life. Adequate nutrition is vital for transition from adolescence to healthy adults as the consequences of malnutrition among children and adolescents include delayed growth, impaired cognitive maturation, lower intellectual quotient, behavioural problems and increased risk of contracting communicable diseases (Mengistu, Alemu, & Destaw, 2013; Onyango, 2013). There are many underlying determinants of undernutrition including poverty, food insecurity, poor sexual and reproductive health, violence, and many infectious and noninfectious diseases (Patton George et al., 2016). The quality of available diets in LMICs is also a challenge as diet is fairly restricted and comprises largely of cereals or legumes with few animal products and a limited access to a variety of fruits and vegetables (Ladipo Oladapo, 2000). Poverty in these settings also leads to limited ability to purchase and consume sufficient amounts of key nutrients. Food insecurity in these settings has also been linked to poor diet quality and uncertainty in the food environment related to inability to access adequate food sources for the sustainability of healthy and active living (Akseer et al. 2017). Food choices and preferences are also determinants of malnutrition since in some settings, despite adequate food access, dietary choices lead to nutritional deficiencies. Adolescents globally are consuming less than adequate amounts of fruits and vegetables and alarmingly high levels of sodium and sugar (Akseer et al. 2017). These poor dietary habits and eating choices pose further threat to the growing bodies. The burden of malnutrition is further complicated for women and girls in LMIC settings owing to the their status and power in society compared to their male counterparts (Jayachandran, 2015).

Micronutrient deficiency is often referred to as hidden hunger and has a global health impact on adolescents because its manifestations are less visible and usually begins to show when the condition is severe and has already led to serious health consequences. A number of nutrition‐specific interventions to address malnutrition have been advocated and these include nutrition education and counselling, micronutrient supplementation, food fortification and macronutrient supplementation.

### 2.2 Description of the intervention

The following interventions (alone or in combination) have been advocated to prevent nutrition deficiencies:

- Nutrition education and counselling
- Micronutrient supplementation and fortification
- Macronutrient supplementation

#### 2.2.1 Nutrition education and counselling

Dietary habits of adolescents are influenced by various factors including food environments, food advertisements, mass media messages, peers and social eating culture (Riebl Shaun et al., 2015; Stang Jamie and Stotmeister, 2017). Nutritional concerns among adolescents include poor dietary habits; low intake of fruits, vegetables, fibre and calcium‐rich foods; high intake of foods high in fat and sugar; unhealthy dieting; and erratic eating behaviours, such as meal skipping (Stang Jamie & Stotmeister, 2017).

Nutrition education and counselling is a widely used strategy to improve nutritional status and change nutrition related behaviours (Story, Lytle Leslie, Birnbaum Amanda, & Perry Cheryl, 2002). The strategy focuses primarily on promoting a healthy diet by increasing the diversity and amount of foods consumed. Nutrition education can help young people attain the knowledge and skills they need to make healthful food choices and develop lifelong healthy eating patterns. Nutrition education and counselling for adolescents have been delivered through various platforms including schools, communities, peer‐based networks and computer and web based education (Kroeze, Werkman, & Brug, 2006; Oenema, Brug, & Lechner, 2001; Pérez‐Rodrigo, & Aranceta, 2001).

#### 2.2.2 Micronutrient supplementation and fortification

Supplementation refers to the provision of individual or mixture of nutrients separately from the diet while adding nutrients to staple foods is termed as fortification. Micronutrients can be supplemented in the form of injections, tablets, capsules, syrups/liquids or powders (Blasbalg Tanya, Wispelwey, & Deckelbaum Richard, 2011). Oral iron supplements, being the most common and inexpensive, have been established as frontline prevention and treatment for iron‐deficiency anaemia (Peyrin‐Biroulet, Williet, & Cacoub, 2015). Other micronutrients most commonly supplemented include calcium, vitamin D, vitamin A, iodine, zinc and MMNs (Haider & Bhutta, 2017; Hess Sonja, Lönnerdal, Christine, Rivera Juan, & Brown Kenneth, 2009; Reid Ian, 2014; Zimmermann & Richard, 2007; Zimmermann Michael & Boelaert, 2015).

Food fortification is the process in which micronutrients are added to processed foods. In many stances, this approach has lead to ameliorating micronutrient deficiencies in the population with reasonable cost making it a very efficient public health intervention. Fortification could be mass fortification (that is adding micronutrients to foods that are commonly consumed such as flour, salt, sugar and cooking oil) or point‐of‐use fortification (that involves adding single‐dose packets of vitamins and minerals in powder form that can be sprinkled onto any ready to eat food consumed at home, school, nurseries, refugee camps or any other place where possible) (WHO, 2014; Zlotkin Stanley et al., 2005).

#### 2.2.3 Macronutrient supplementation

Macronutrient interventions include supplementary feeding, balanced energy and protein supplementation and lipid based nutrition supplementation (LNS). Supplementary feeding is the provision of extra food to children or families beyond the normal ration of their home diets, and can take place in homes, feeding centres, healthcare centres and schools (Sguassero, de Onis, Bonotti Ana, & Carroli, 2012). Energy protein supplements are used to increase the total daily protein and calorie intake in order to aid nutrition and it involves supplements in which protein provides <25% of the total energy content. These are available in both oral and parenteral form. Oral supplements could be in the form of whole protein milk and beverages. These supplements also contain a wide range of micronutrients which may benefit the consumer. LNS are a family of products in which majority of the energy is from lipids; they also include protein and essential fatty acids and a range of micronutrients (Dewey Kathryn, & Arimond, 2012).

### 2.3 How the intervention might work

#### 2.3.1 Nutrition education and counselling

Nutritional concerns among the adolescent age group make them vulnerable to environmental influences and consequent unhealthy eating behaviours (Riebl Shaun et al., 2015; Stang Jamie & Stotmeister, 2017). Therefore, promotion of healthy nutrition during adolescence is vital to inculcate sustainable healthy dietary habits. Nutrition education and counselling at this stage can create knowledge through active, fun and interactive processes and promote behaviour changes in food attitudes and practices (Baldasso, Galante Andrea, & De Piano Ganen, 2016). Such programmes can increase adolescents' ability to understand proper food practices and encourage them to actively adopt healthy food habits. It is important to note that nutrition education and counselling alone have higher chances of success if there are no other serious constraining factors in terms of access to foods and the intervention is appropriately designed for the target population group (Harrison, 2010). There is some evidence that in relatively advantaged populations, targeted educational approaches can work well (Contento et al., 1995; Harrison, 2010). If provided under ideal circumstances, nutrition education and counselling have the potential to address multiple nutrient deficiencies without the risks of toxicity and interactions.

#### 2.3.2 Micronutrient supplementation and fortification

Direct supplementation of vulnerable subpopulations with micronutrients, usually through a primary healthcare system or healthcare delivery system such as an immunisation programme, has been shown to be effective and cost‐effective. A direct supplementation approach through a healthcare delivery system has the advantage of directly reaching portions of the population most at risk while not putting other segments of the population at risk of over consumption or adverse interactions (Harrison, 2010). The long‐term disadvantages, however, relate primarily to sustainability, coverage and compliance. Supplementation depends upon a viable delivery system with built‐in quality control, as well as wide coverage and high uptake rates among vulnerable individuals and families. Supplementation only works if the supplements are available and accessible and the intended individuals actually take them. The risks of using dietary supplements might include organ damage from inherent toxicity, interactions or product contamination (Harrison, 2010).

The advantage of food fortification, provided that safe and effective levels of the relevant nutrients can be delivered through an appropriate food vehicle, is that no or minimal behaviour change is required on the part of the population. This provides a tremendous advantage in terms of coverage and efficiency. Food fortification adopts an integrated approach and provides support to improve micronutrients malnutrition when other existing food supplies fail to do so (Allen, De Benoist, Dary, Hurrell, & World Health Organization, 2006).

#### 2.3.3 Macronutrient supplementation

Supplementary feeding, balanced energy and protein supplementation and LNS are designed to increase the total daily protein and calorie intake in order to aid nutrition (Sguassero et al., 2012). Supplementary feeding can improve the quality and quantity of the daily nutritional intake by providing additional calories, minerals and vitamins consequently leading to better nutritional status, however there are issues of compliance, improving coverage and sustainability. Although food supplementation can aid in improving current nutritional situation, it is not a solution to the primary health and nutritional problems faced by families living in poverty. Macronutrient interventions have many of the same problems as micronutrient interventions including sustainability, coverage and compliance.

We aim to assess the impact of these interventions alone or in combination on adolescent health and nutrition status in LMIC. There is an increasing evidence that health initiatives require health systems that can deliver services equitably and efficiently; and thus, many global health initiatives now involve health systems strengthening measures into their programmes (WHO, 2010). Therefore, we also aim to assess various health system components using the World Health Organisation (WHO) health system building blocks framework (WHO, 2010). This will aid the understanding of how these areas are utilised in planning and delivering equitable and contextually appropriate nutrition interventions for adolescents.

### 2.4 Why it is important to do this review

Malnutrition is one of the most common causes of morbidity and mortality among children and adolescent population worldwide (UNICEF, 2005); half of the global child mortality is attributable to malnutrition (IGME, 2017). With about one quarter of the total world population (1.8 billion people) comprising adolescents and young adults (Ameratunga, 2017; UNPFA, 2014); it has become even more important to identify effective interventions targeting adolescents to improve their health and nutrition status to ensure sustainable healthy behaviours along with healthy growth and development (Sawyer Susan et al., 2012).

Globally, there is an increased focus on adolescents and youth as reflected by the sustainable development goals. Existing systematic reviews assessing the impact of nutrition interventions among adolescents are either not comprehensive (assessing a single intervention or a specific micronutrient); have overlapping age groups (includes children and youth along with adolescents); or are focused on female adolescents only (Lassi Zohra, Anoosh, Das Jai, Salam Rehana, & Bhutta Zulfiqar, 2017; Salam Rehana et al., 2016). The majority of the existing systematic reviews have restricted their included studies to randomised trials without focusing on various contextual factors that might potentially impact the effect of nutrition interventions in this age group. Moreover, the impact of nutrition education and counselling in this age group has not been systematically reviewed. Table 1 describes the existing systematic reviews.

This review aims to comprehensively evaluate the effectiveness of all the above mentioned preventive nutrition interventions in combination or alone. We aim to include large‐scale programme evaluations that are implemented in multiple communities targeting adolescents with the above mentioned nutrition interventions. We also aim to assess various contextual factors that might potentially influence the effectiveness of these nutrition interventions in this age group. This contextual information will be based on the WHO health system building blocks framework describing health systems in terms of six core components: service delivery, health workforce, health information systems, access to essential medicines/supplies, financing and leadership/governance (WHO, 2010). Findings from this review will assist the policy makers in designing contextually appropriate nutrition intervention initiatives targeting this important age group.

## 3 OBJECTIVES

The objective of this review is to assess the impact of preventive nutrition interventions (including nutrition education and counselling, micronutrient supplementation/fortification and macronutrient supplementation) to improve the health and nutritional status of adolescents aged 10–19 years of age in LMICs.

The secondary objective of the this review is to assess the various contextual factors based on the WHO health system building blocks framework that might potentially impact the effectiveness of these interventions in this age group.

## 4 METHODS

### 4.1 Criteria for considering studies for this review

#### 4.1.1 Types of studies

We included primary studies, including large‐scale programme evaluations, using experimental and quasiexperimental study designs. The following study designs were eligible for inclusion:

- Randomised controlled trials including both cluster and individual level randomisation
- Quasiexperimental studies with nonrandom assignment to intervention and comparison groups
- Controlled before‐after studies in which observations are made before and after the implementation of an intervention, both in a group that receives the intervention and in a control group that does not.
- Interrupted time series studies that uses observations taken at least three time points before and after an intervention to detect whether the intervention has had an effect significantly greater than any underlying trend over time.

We intended to include quasiexperimental study designs, such as CBA and ITS, along with RCTs since we intended to assess the effectiveness of large scale programme evaluations that might not have been conducted in a randomised design. Moreover, we also intended to assess various contextual factors based on the WHO health system building blocks as they could potentially impact the uptake and effectiveness of these interventions.

#### 4.1.2 Types of participants

The target population was adolescents between 10 and 19 years of age from LMICs. We classified LMIC according to the World Bank criteria (World Bank). We excluded studies conducted specifically among hospitalised adolescents and adolescents with any pre‐existing health conditions. Studies including only a subset of eligible participants were included only if the results provided information for the relevant subgroup separately.

#### 4.1.3 Types of interventions

The following interventions alone or in any combination were reviewed:

- Nutrition education and counselling (provision of general information related to health with or without nutrition assessment, identification of individual nutrition needs and goals and discussing ways to meet those goals provided in any setting)
- Micronutrient supplementation and fortification (any micronutrient alone or in combination)
- Macronutrients supplementation

We analysed different individual interventions separately and studies assessing a combination of interventions were also analysed separately. Eligible comparisons were no intervention or placebo (whatever was applicable in the setting where study was conducted).

#### 4.1.4 Types of outcome measures

We included all of the studies that met our inclusion criteria, but only those studies that had the outcomes defined below were included in the meta‐analysis.

##### 4.1.4.1 Primary outcomes

- Anaemia (haemoglobin concentrations <12 g/dL)
- Body mass index (defined as weight in kg divided by height in metres squared)
- Morbidity (any morbidity as reported by the study authors for, e.g., infectious diseases, night blindness, etc.)
- Adverse effects (as reported by study authors)

##### 4.1.4.2 Secondary outcomes

- Haemoglobin concentration (measured in any units)
- Micronutrient status (measured in any units)
- Body composition (measured in any units)
- Development outcomes (as reported by authors; could include cognitive development, interpersonal development and social development)
- All‐cause mortality

##### 4.1.4.3 Duration of follow‐up

We included studies with any duration of follow‐up.

##### 4.1.4.4 Type of settings

We included studies conducted in community, facility or school settings in LMICs.

### 4.2 Search methods for identification of studies

#### 4.2.1 Electronic searches

The search was performed till February 5, 2019 in the following electronic databases:

- Cochrane Controlled Trials Register (CENTRAL) (CENTRAL; 2019) (searched February 5, 2019)
- MEDLINE (searched from 1946 to February 7, 2019)
- EMBASE (searched from 1974 to February 6, 2019)
- CINAHL (searched from 1937 to February 8, 2019)
- PsycINFO (searched February 9, 2019)
- the WHO nutrition databases (http://www.who.int/nutrition/databases/en/) (searched February 9, 2019)
- CAB Global Health (searched February 9, 2019)
- Social Science Citation Index (searched from 1970 to February 10, 2019)
- Scopus (searched February 10, 2019)
- WHO Global Health Index (searched February 9, 2019)
- ADOLEC (http://bases.bireme.br/cgi‐bin/wxislind.exe/iah/adolec/?IsisScript=iah/iah.xis&base=ADOLEC&lang=i&form=A) (searched February 10, 2019)
- EPPI (http://bases.bireme.br/cgi‐bin/wxislind.exe/iah/adolec/?IsisScript=iah/iah.xis&base=ADOLEC&lang=i&form=A) (searched February 10, 2019)

The trials registry Clinicaltrials.gov was searched for ongoing trials. We searched Google Scholar along with key nutrition agencies database such as Nutrition International (https://www.nutritionintl.org/), the Global Alliance for Improved Nutrition (https://www.gainhealth.org/homepage), the World Food Programme (https://www.wfp.org/) and HarvestPlus (https://www.harvestplus.org/) to search for nonindexed, grey literature to locate relevant programme evaluations and any additional trials. We did not apply any restrictions based on publication date, language or publication status. Search strategies for MEDLINE, CENTRAL and CINAHL is added as Appendix 1; we used the same search strategy for other search engines.

#### 4.2.2 Searching other resources

We made every effort to contact relevant organisations and experts in the field to identify unpublished or ongoing studies. We also searched Eldis.org to find organisations with an interest in nutrition. References of included articles, relevant reviews and annotated bibliographies were scanned for eligible studies. We conducted forward citation searching of included studies in Google Scholar to identify any recent studies missed from the database searches.

### 4.3 Data collection and analysis

#### 4.3.1 Selection of studies

Two reviewers (O. I. and W. A.) independently screened titles and abstracts in duplicate. We pilot‐tested the screening criteria at both title and abstract screening stage and full text stage. We used the PRISMA flow diagram to report eligibility of studies. We retrieved the full text of all studies which passed this first level screening. The full text review were also done in duplicate by two reviewers, and agreement was reached by consensus. Disagreements were resolved by consultation with a third reviewer (S. S.). We collated multiple reports of the same study, so that each study rather than each report was the unit of interest in the review. We examined any relevant retraction statements and errata for information.

#### 4.3.2 Details of study coding categories

Two review authors (R. A. S. and O. I.) extracted data independently and a third review author (J. K. D.) checked for reliability and resolved any conflict. We extracted the primary data for the study characteristics including details of the populations, setting, sociodemographic characteristics, interventions, comparators, outcomes and study design in duplicate. Disagreements were resolved by discussion or consultation with a third reviewer.

The following information was extracted for each included study:

- Background: time period when study took place, type of publication (e.g., full‐text journal article, abstract, conference paper, thesis), study country or countries
- Population and setting: population age and setting
- Methods: Study design, description of study arms, unit of allocation, sample or cluster size per study arm (for individually or cluster randomised trials respectively), start and end date, follow up
- Participants: total number randomised/allocated, sample representativeness, baseline characteristics, number of withdrawals, sociodemographic data
- Intervention group details: number randomised/allocated to group, description of intervention, duration and follow‐up, timing, delivery of intervention, providers and their training. We described all the study intervention arms in the tables of included studies, however, we only reported the intervention arms that met review inclusion criteria.
- Comparison group details: number randomised to group, description of comparison, duration and follow‐up, timing, providers and their training
- Outcomes: measurement tool, validation of the tool, total number in intervention and comparison groups, change indicated at each time point
- Other information: study start date, study end date, funding sources and conflict of interest.

In addition to the above mentioned details, we also collected details related to the programme related contextual factors. This information was based on the WHO health system building blocks framework describing health systems in terms of six core components (WHO, 2010):

- Service delivery: The availability of health services including all services dealing with the delivery of nutrition interventions.
- Health workforce: The availability of sufficient and capable staff to deliver nutrition interventions.
- Health information systems: The availability of the production, analysis, dissemination and use of reliable and timely information on health and nutrition related determinants and status.
- Access to essential medicines/supplies: The availability of nutrition intervention related commodities and supplies in adequate amounts, in the appropriate dosages and at an affordable price.
- Financing: The sources of funds available for the delivery of nutrition interventions.
- Leadership/governance: The roles and responsibilities of various sectors including public, private and voluntary sectors in implementing the nutrition interventions.

#### 4.3.3 Assessment of risk of bias in included studies

For RCTs we used the Cochrane risk of bias tool (Higgins & Green, 2011) which assesses selection bias, performance bias, detection bias, attrition bias and reporting bias. We rated each component as “high”, “low” or “unclear” for each risk of bias component. For nonrandomised studies, we used the Cochrane Effective Practice and Organisation of Care (EPOC) risk of bias criteria (based on additional criteria including similar baseline outcome measurements, similar baseline characteristics, knowledge of the allocated interventions adequately prevented during the study, protection against contamination, intervention independent of other changes, shape of intervention effect prespecified and intervention unlikely to affect data collection) and rated the studies as low risk, high risk or unclear risk (EPOC, 2017). We provided supporting evidence for the risk of bias judgements.Two independent reviewers performed quality appraisal for each study and disagreements were resolved by discussion or consultation with a third reviewer. We summarised the quality of evidence according to the outcomes as per the Grading of Recommendations, Assessment, Development and Evaluation (GRADE) criteria (Walker, Fischer‐Walker, Bryce, Bahl, & Cousens, 2010). A grade of “high”, “moderate”, “low” and “very low” was used for grading the overall evidence indicating the strength of an effect on specific health outcome based on methodological flaws within the component studies, consistency of results across different studies, generalisability of research results to the wider patient base and how effective the treatments have shown to be (Balshem et al., 2011). For nonrandomised studies, the evidence quality was upgraded based on large magnitude of effect, dose‐response relationship and effect of all plausible confounding factors would be to reduce the effect (where an effect is observed) or suggest a spurious effect (when no effect is observed). Two reviewers discussed ratings and reached consensus, and disagreements were resolved by consulting a third reviewer. We developed a summary of findings table to show the effects for the primary outcomes.

#### 4.3.4 Synthesis procedures and statistical analysis

The following synthesis procedures and analysis methods were used:

#### 4.3.5 Measures of treatment effect

We performed statistical analysis using RevMan 5 (Revman, 2014). For dichotomous data, we used odds ratios (OR), and risk ratios (RR) with 95% confidence intervals (CI). For continuous data, we used the mean difference (MD) with 95% CI, if outcomes were measured in the same way between trials. We used the standardised mean difference (SMD) with 95% CI to combine trials that measured the same outcome but used different methods of measurement.

#### 4.3.6 Unit of analysis issues

Before initiating the synthesis, we ensured that all articles reporting on the same study were appropriately linked. To ensure independence and appropriate combination of outcome constructs, we synthesised the data according to the type of interventions specified above. If multiarm studies were included, we combined intervention groups or separated into different forest plots, and ensured that there was no double counting of participants. If an outcome was reported in several different metrics, we performed unit conversions in order to pool the data. We anticipated differences in the types of literature and ensured that any analysis take possible sources of dependency into account by grouping papers into studies and ensuring that no double counting of evidence took place when synthesising across studies.

Two trials (Agarwal, Gomber, Bisht, & Som, 2003; Zhu et al., 2005) reported the outcomes of interest at multiple time points, we coded the data for outcomes from all reported time points and then reported the the outcomes from the time point closest to other studies. Where trials used clustered randomisation, we anticipated that study investigators would have presented their results after appropriately controlling for clustering effects (e.g., variance inflated standard errors, hierarchical linear models). If it was unclear whether a cluster‐RCT had appropriately accounted for clustering, we planned to contact the study investigators for further information. Where appropriate controls for clustering were not used, we requested an estimate of the intra‐class correlation coefficient. We used the “inflated standard error” approach to calculate the correct estimates by multiplying the standard error with the square root of the design effect (Higgins, Altman, & Sterne, 2011a).

#### 4.3.7 Dealing with missing data

If the outcome of interest did not include data on all participants, we first contacted the study authors via email to inquire about data for the missing cases. Missing data, if found, were reincluded in the analysis. If we were unable to find the missing data, we analysed data for only those participants whose results were available, and addressed the impact of the missing data in the assessment of risk of bias. Only one study (Sen, 2009) had high attrition (29% loss to follow‐up) and we analysed data for only those participants whose results were available.

#### 4.3.8 Assessment of heterogeneity

We assessed heterogeneity among studies in two ways. Firstly, we assessed heterogeneity at face value: heterogeneity in population, interventions, or outcomes. We used *I* ^2^, *Q* and *τ* ^2^ statistics as a guide to assess heterogeneity along with a visual inspection of forest plots.

#### 4.3.9 Assessment of reporting biases

There were only nine studies included in this review; therefore we could not assess for the reporting bias. For future updates funnel plots would be used if there are 10 or more studies in meta analysis for one outcome and investigation will be conducted for reporting biases, for example, publication bias.

#### 4.3.10 Data synthesis

A meta‐analysis was conducted separately for each outcome and intervention. Furthermore, for each outcome, we separately meta‐analysed different study designs (RCT, ITS and CBA). We pooled data from studies we judged to be clinically homogeneous, if more than one study provided usable data in any single comparison, we performed a meta‐analysis. We standardised all the reported effect sizes as RRs for the dichotomous outcome and MDs or SMDs for the continuous outcomes. We attempted to standardise the outcomes as a common metric and synthesised together, where possible. We carried out statistical analysis using the Review Manager software (Revman, 2014). We used random‐effects meta‐analysis for combining data to produce an overall summary, since we expected reasonable clinical heterogeneity in interventions, comparisons, outcomes and settings within the studies included. The random‐effects summary was treated as the average of the range of possible treatment effects and we discussed the clinical implications of treatment effects differing between trials. We reported statistical heterogeneity as *I* ^2^, *Q* and *τ* ^2^ statistics for all random‐effects meta‐analyses. We narratively synthesised and reported the findings on the contextual factors based on the WHO health system building blocks framework for each intervention.

#### 4.3.11 Subgroup analysis and investigation of heterogeneity

Based on the availability of the data, we had planned to conduct subgroup analysis for following subgroups:

- Duration or intensity of intervention (e.g., short vs. long term, one‐off vs. multiple sessions).
- Individual context versus group context (for nutrition education and counselling only, that is, children receiving the intervention individually vs. those in groups)
- Study setting: school, community, clinic, and so forth.
- Sex: Male and females.
- Population (e.g., urban population vs. rural population; resource poor vs. resource rich population)
- We also attempted to conduct subgroup analysis based on the WHO health system building blocks factors (where data was available).

However, since very few studies were included in each comparison within the review, we could not conduct any of the afore mentioned subgroup analysis. We did, however, subgrouped the outcomes according to the specific micronutrients being supplement under the comparison of “Micronutrient Supplementation/Fortification” for clarity. For future updates, we plan to assess difference in subgroups based on the methodology described in the Cochrane Handbook (Higgins & Green, 2011) by using a simple approach for a significance test to investigate differences between two or more subgroups. We will undertake a standard test for heterogeneity across subgroup results using *χ* ^2^ test or moderator analysis rather than across individual study results.

#### 4.3.12 Sensitivity analysis

We had planned to conduct sensitivity analyses to consider the impact of the following:

- Allocation concealment (adequate vs. inadequate and/or unclear).
- Attrition (< 20% vs. ≥20%).

However, since very few studies were included in the review, we could not conduct any sensitivity analysis.

#### 4.3.13 Treatment of qualitative research

We did not include qualitative studies.

## 5 RESULTS

### 5.1 Description of studies

See Characteristics of included studies; Characteristics of excluded studies; Characteristics of studies awaiting classification.

#### 5.1.1 Results of the search

**Figure 1:**
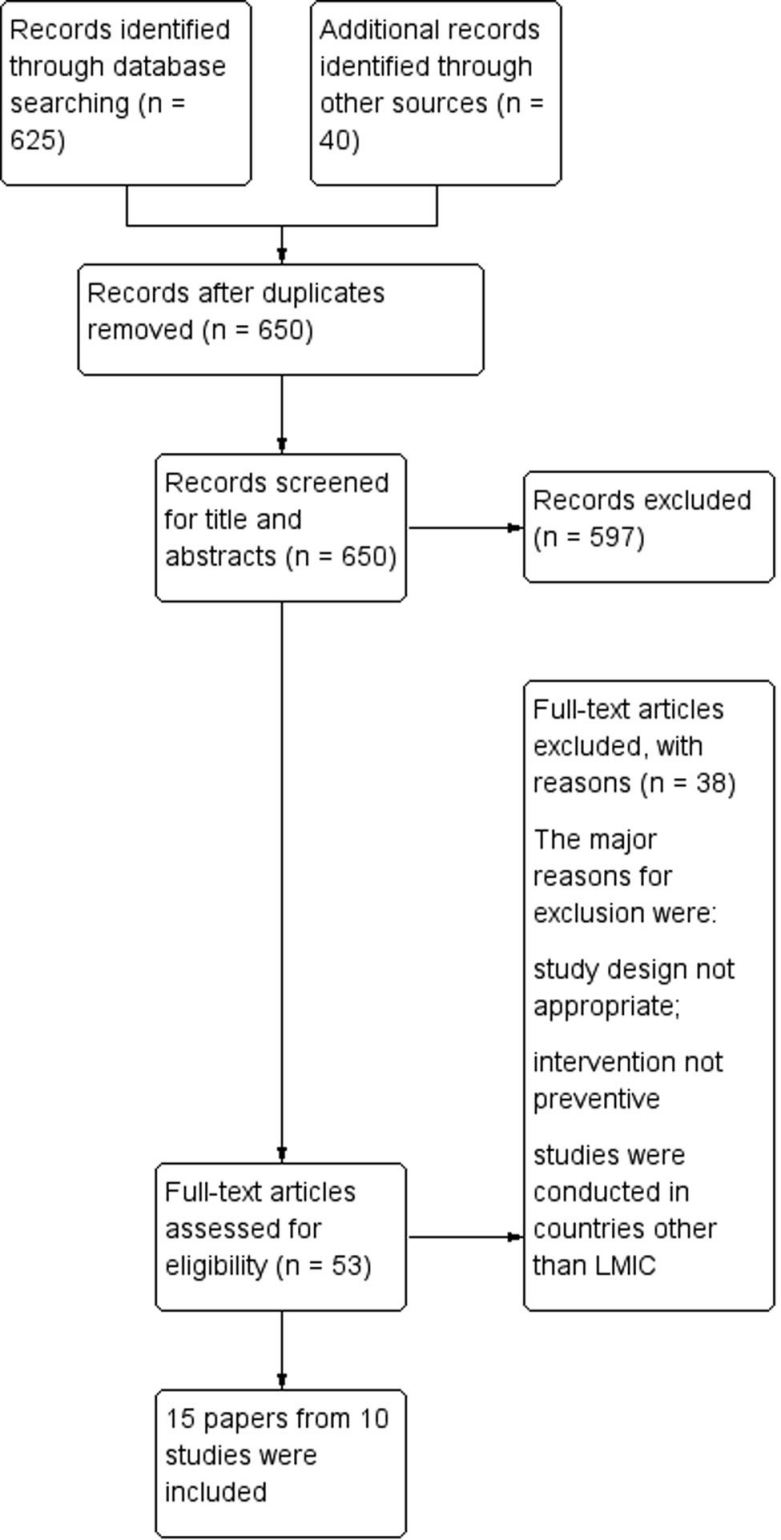
Study flow diagram

We identified a total of 665 potentially relevant titles from the search. After removing duplicates, we screened 650 records for eligibility and excluded 597 articles on the basis of titles and abstracts. We obtained the full‐text reports of the remaining 53 records, and of these, excluded 38 and included 15 papers (10 studies) in the review. Figure 1 depicts the search flow diagram.

#### 5.1.2 Included studies

This review includes 15 papers from 10 studies including 10,802 participants (Agarwal et al., 2003; Chiplonkar & Kawade, 2012; Februhartanty, Dillon, & Khusun, 2002; Goyle, 2012; Hettiarachchi, Liyanage, Wickremasinghe, Hilmers, & Abrams, 2008; Hyder et al., 2007; Khadilkar et al., 2010; Sen, 2009; Soekarjo et al., 2004; Zhu et al., 2005). All the studies were RCTs.

##### 5.1.2.1 Settings

All of the studies were conducted between 2003 and 2012 in LMICs including China (Zhu et al., 2005), India (Agarwal et al., 2003; Chiplonkar & Kawade, 2012; Goyle, 2012; Khadilkar et al., 2010; Sen, 2009), Sri Lanka (Hettiarachchi et al., 2008), Bangladesh (Hyder et al., 2007) and Indonesia (Februhartanty et al., 2002; Soekarjo et al., 2004). These studies were all conducted in school settings.

##### 5.1.2.2 Participants

The majority of the studies (eight out of 10 studies) included adolescent girls aged between 10 and 19 years of age. Hettiarachchi et al. (2008) included both female and male adolescents from 12 to 16 years of age. Soekarjo et al. (2004) included both adolescent girls and boys aged 12–15 years. Zhu et al. (2005) was conducted among girls aged 10–12 years of age; Khadilkar et al. (2010) included girls 14 to 15 years of age; Sen (2009) included girls 9–13 years of age; Agarwal et al. (2003) included girls 10–17 years of age; Chiplonkar and Kawade (2012) and Goyle (2012) included girls 10–16 years of age. Two studies mentioned that the participants were adolescent girls but did not specify the age group; the mean age of adolescent girls in Hyder et al. (2007) was 12 years; while Februhartanty et al. (2002) included postmenarchal female adolescent girls with mean age 14.6 years.

##### 5.1.2.3 Interventions

We did not find any study assessing nutrition education and counselling or macronutrient supplementation. All of the included studies provided micronutrient supplementation/fortification (any micronutrient alone or in combination). Among the micronutrient supplementation/fortification studies; two studies (Khadilkar et al., 2010; Zhu et al., 2005) provided calcium/vitamin D supplementation/fortification; five studies (Agarwal et al., 2003; Februhartanty et al., 2002; Hettiarachchi et al., 2008; Sen, 2009; Soekarjo et al., 2004) provided iron supplementation with or without folic acid; two studies (Chiplonkar & Kawade, 2012; Hettiarachchi et al., 2008) provided zinc supplementation; one study (Soekarjo et al., 2004) provided vitamin A supplementation and three studies assessed MMN fortification (Chiplonkar & Kawade, 2012; Goyle 2012; Hyder et al., 2007). The duration of intervention ranged from a minimum of 10 weeks supplementation (Chiplonkar & Kawade, 2012) to a maximum of 2 years of intervention (Zhu et al., 2005).

Three of the studies had multiple intervention arms:

- Chiplonkar and Kawade (2012) provided MMN fortified snack in one group and zinc supplement in the other group
- Hettiarachchi et al. (2008) provided iron supplement in one group and zinc supplement in the other group
- Soekarjo et al. (2004) provided iron and folate supplement in one group, vitamin A supplement in one group and iron, folate and vitamin A together in one group.

We have reported the data from the relevant intervention arm under their respective intervention subgroups.

##### 5.1.2.4 Outcomes

Among primary outcomes, included studies reported anaemia and BMI. Among secondary outcomes, haemoglobin concentrations, micronutrient status (zinc, vitamin A and vitamin D levels), body composition (total body BMC and total body BMD) and developmental outcomes were reported. None of the included studies reported morbidity and adverse effects among the primary outcomes and all‐cause mortality among the secondary outcomes.

We could not pool the outcomes for one study since it reported outcomes for prepubertal and post pubertal girls and boys separately for all the intervention arms and hence we have narratively reported the findings from this study under the specific outcomes.

##### 5.1.2.5 Contextual factors based on the WHO health system building blocks framework

All the included studies were RCT and we did not find any large scale nutrition intervention programmes targeting adolescents from LMICs. We have narratively synthesised the findings from the six health system building blocks based on WHO health system building blocks framework (Table 2):

###### 5.1.2.5.1 Service delivery

The service delivery platform in all of the included studies was school and the nutrition intervention in each study was delivered in school.

###### 5.1.2.5.2 Health workforce

The nutrition interventions in Februhartanty et al. (2002), Hettiarachchi et al. (2008), Hyder et al. (2007), Khadilkar et al. (2010) and Sen (2009) were delivered through school teachers and student class monitors working with the study investigators. In Soekarjo et al. (2004), the intervention was delivered through field workers. Agarwal et al. (2003), Chiplonkar and Kawade (2012), Goyle (2012) and Zhu et al. (2005) did not clearly specify the workforce utilised for the nutrition intervention delivery; however from the description it appeared that the intervention was probably delivered through school teachers.

###### 5.1.2.5.3 Health information system

None of the included studies specified the details pertaining to health information systems.

###### 5.1.2.5.4 Access to essential medicines/supplies

In all of the included studies, the nutrition supplement was provided by the researcher.

###### 5.1.2.5.5 Financing

Financing was provided by various not‐for‐profit organisations including UNICEF, Micronutrient Initiative, Zensar Foundation, SEAMEO‐TROPMED Regional Center for Community Nutrition, University Grants Commission, International Atomic Energy Agency, Australian Dairy Research and Development Corporation and Murray Goulburn Co‐operative Co. Khadilkar et al. (2010) did not specify the financing while there was no funding for Sen 2009.

###### 5.1.2.5.6 Leadership/governance

In all of the included studies, study investigators led the intervention.

##### 5.1.2.6 Clustering

Three of the included studies were cRCTs (Agarwal et al., 2003; Sen, 2009; Soekarjo et al., 2004). We used appropriate cluster adjusted estimates as specified in the “Unit of analysis issues” section of the methodology to adjust for clustering in both the cRCTs.

#### 5.1.3 Excluded studies

We excluded 38 studies (Abrams et al., 2005; Angeles‐Agdeppa et al., 1997; Beasley et al., 2000; Castillo‐Durán, Marín, Alcázar, Iturralde, & Ruz, 2001; Chan, McElligott, McNaught, & Gill, 2006; Damsgaard, Mølgaard, Matthiessen, Gyldenløve, & Lauritzen, 2012; De Oliveiera, 2009; Ahmed et al., 2005, 2010; Deshmukh, Garg, & Bharambe, 2008; Diogenes et al., 2013; Dongre, Deshmukh, & Garg, 2011; Eftekhari et al., 2006; Friis et al., 1997; Ganmaa et al., 2017; Ilich‐Ernst et al., 1998; Kianfar, Kimiagar, & Ghaffarpour, 2000; Kotecha, Nirupam, & Karkar, 2009; Lambert, Eastell, Karnik, Russell, & Barker, 2008; Ma, Huang, Yang, & Su, 2014; Manger et al., 2008; Mann, Kaur, & Bains, 2002; McKenna, Ilich, Andon, Wang, & Matkovic, 1997; Mwaniki et al., 2002; Pilz, Hahn, Schön, Wilhelm, & Obeid, 2017; Prentice et al., 2005; Prentice, Dibba, Sawo, & Cole, 2012; Rerksuppaphol & Rerksuppaphol, 2016; Rousham et al., 2013; Sarma, Udaykumar, Balakrishna, Vijayaraghavan, & Sivakumar, 2006; Schou, Heuck, & Wolthers, 2003; Shah & Gupta, 2002; Silk, Greene, Baker, & Jander, 2015; Sunawang, Hidayat, & Kusharisupeni, 2009; Tee et al., 1999; Viljakainen et al., 2006; White, Cox, Peters, Pipingas, & Scholey, 2015; Yusoff, Wan Daud, & Ahmad, 2012).

Out of these 38 studies, participants in four studies (Manger et al., 2008; Prentice et al. 2012; Rerksuppaphol & Rerksuppaphol, 2016; Sarma et al., 2006) included both children and adolescents. We wrote emails to these four authors to obtain data for the adolescent subgroup. We received response from Manger et al. 2008 stating that the number of adolescents was too small while three (Prentice et al. 2012; Rerksuppaphol & Rerksuppaphol, 2016; Sarma et al., 2006) of the other authors did not respond to the emails and hence these studies were excluded from the review.

The major reasons for exclusion were that the study design was not appropriate; the intervention was therapeutic and/or that the studies were conducted in countries other than LMIC. Please see Characteristics of excluded studies.

### 5.2 Risk of bias in included studies

**Figure 2:**
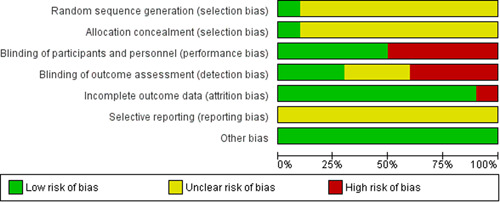
Risk of bias graph: review authors' judgements about each risk of bias item presented as percentages across all included studies

**Figure 3:**
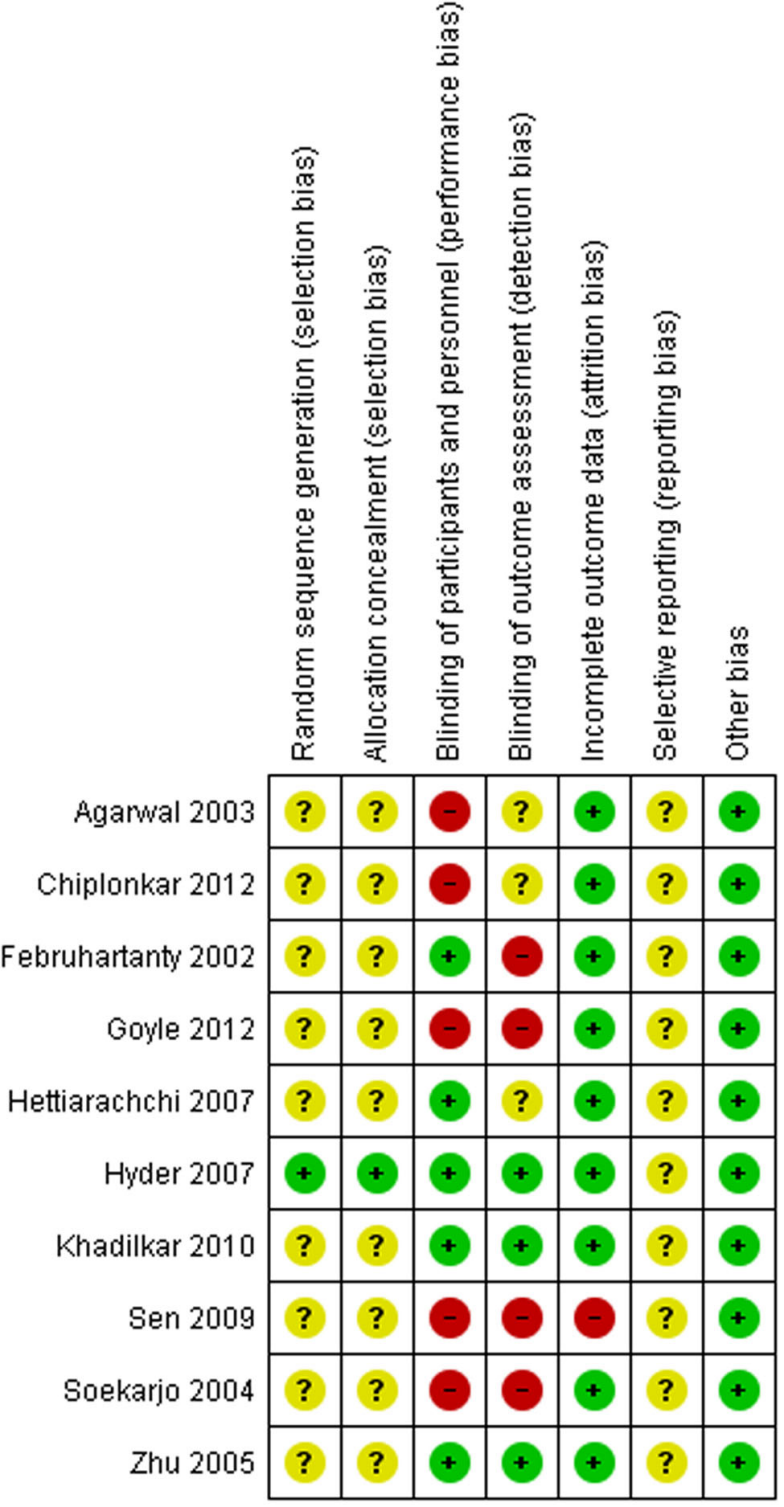
Risk of bias summary: review authors' judgements about each risk of bias item for each included study

Overall the included studies were judged to be at unclear risk of bias due to insufficient information regarding sequence generation and allocation concealment. The majority of the studies lacked blinding and were judged to be at high risk or unclear risk for blinding. The majority of the studies were at low risk of bias for incomplete outcome data, selective reporting and other biases. The summary of the risk of bias across the included studies is shown in Figures 2 and 3.

#### 5.2.1 Allocation (selection bias)

Only one study (Hyder et al., 2007) was judged to be at low risk of bias for sequence generation and allocation concealment. All other studies (Agarwal et al., 2003; Chiplonkar & Kawade, 2012; Februhartanty et al., 2002; Goyle, 2012; Hettiarachchi et al., 2008; Khadilkar et al., 2010; Sen, 2009; Soekarjo et al., 2004; Zhu et al., 2005) were judged to be at unclear risk of bis due to insufficient information regarding the methods for sequence generation and allocation concealment.

#### 5.2.2 Blinding (performance bias and detection bias)

For the blinding of participants and personnel, five studies (Februhartanty et al., 2002; Hettiarachchi et al., 2008; Hyder et al., 2007; Khadilkar et al., 2010; Zhu et al., 2005) were judged to be at low risk of bias for blinding of participants and personnel; while five studies (Agarwal et al., 2003; Chiplonkar & Kawade, 2012; Goyle, 2012; Sen, 2009; Soekarjo et al., 2004) were rated to be at high risk due to lack of blinding of participants and personnel.

For blinding of outcome assessors, three studies (Hyder et al., 2007; Khadilkar et al., 2010; Zhu et al., 2005) were judged to be at low risk of bias, three studies (Agarwal et al., 2003; Chiplonkar & Kawade, 2012; Hettiarachchi et al., 2008) were rated to have unclear risk of bias, while four studies (Februhartanty et al., 2002; Goyle, 2012; Sen, 2009; Soekarjo et al., 2004) were rated to be at high risk of bias due to absence of blinding of the outcome assessors.

#### 5.2.3 Incomplete outcome data (attrition bias)

All studies except one (Sen, 2009) were judged to be at a low risk of attrition bias. Sen (2009) had about 30% overall loss to follow‐up rate.

#### 5.2.4 Selective reporting (reporting bias)

None of the included studies mentioned information regarding trial registration and we did not find any prior published protocol for any of the included studies. The studies were judged to be at low risk of selective reporting since the outcomes specified in the methodology section have been reported in the results section.

#### 5.2.5 Other potential sources of bias

Two studies (Agarwal et al., 2003; Goyle, 2012) did not specify sample size assumptions. There was no other bias detected in any of the other included studies.

### 5.3 Effects of interventions

#### 5.3.1 Comparison 1: Nutrition education and counselling

We did not find any study assessing the impact of nutritional education and counselling on health and nutritional status among adolescents in LMICs.

#### 5.3.2 Comparison 2: Micronutrient supplementation and fortification (any micronutrient alone or in combination)

A total of 15 papers from 10 studies including 10,802 participants assessed the impact of micronutrient supplementation/fortification. Two studies (Khadilkar et al., 2010; Zhu et al., 2005) assessed calcium/vitamin D supplementation/fortification; four studies (Agarwal et al., 2003; Februhartanty et al., 2002; Hettiarachchi et al., 2008; Sen, 2009) assessed iron supplementation with or without folic acid; two studies (Chiplonkar & Kawade, 2012; Hettiarachchi et al., 2008) assessed zinc supplementation; while three studies assessed MMN fortification (Chiplonkar & Kawade, 2012; Goyle, 2012; Hyder et al., 2007). Two of the studies had multiple intervention arms and were included in multiple comparison groups. Chiplonkar and Kawade (2012) provided MMN fortified snack in one group and zinc supplement in the other group while Hettiarachchi et al. (2008) provided iron supplement in one group while zinc supplement in the other group.

##### 5.3.2.1 Primary outcomes

Among the primary outcomes, included studies reported anaemia and BMI. None of the included studies reported on any other primary outcome, including morbidity or adverse effects.

###### 5.3.2.1.1 Anaemia: Single study result

**Figure 4:**
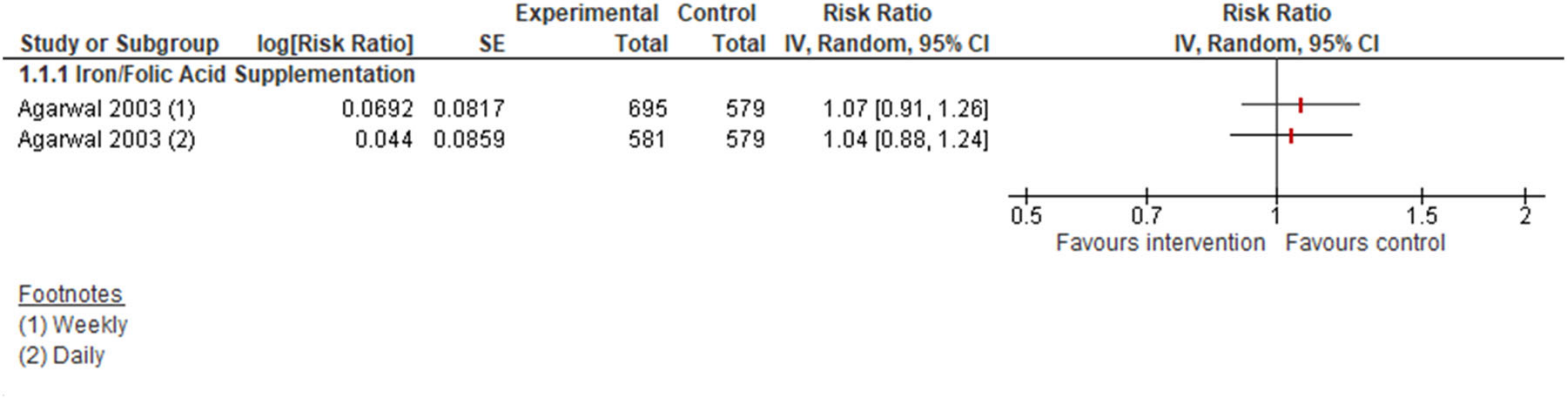
(Analysis 1.1) Forest plot of comparison: 1 Micronutrient Supplementation/Fortification versus No Supplementation/Fortificaton, outcome: 1.1 Anaemia

One study (Agarwal et al., 2003) reported on anaemia. We are uncertain of the effect of iron supplementation with or without folic acid among adolescents on anaemia.(daily supplementation RR: 1.04, 95% CI: 0.88, 1.24; one study; 1,160 participants; low quality evidence; Analysis 1.1; weekly supplementation RR: 1.07, 95% CI: 0.91, 1.26; one study; 1,247 participants; very low quality evidence; Analysis 1.1; Figure 4).

###### 5.3.2.1.2 BMI: Pooled study result

**Figure 5:**
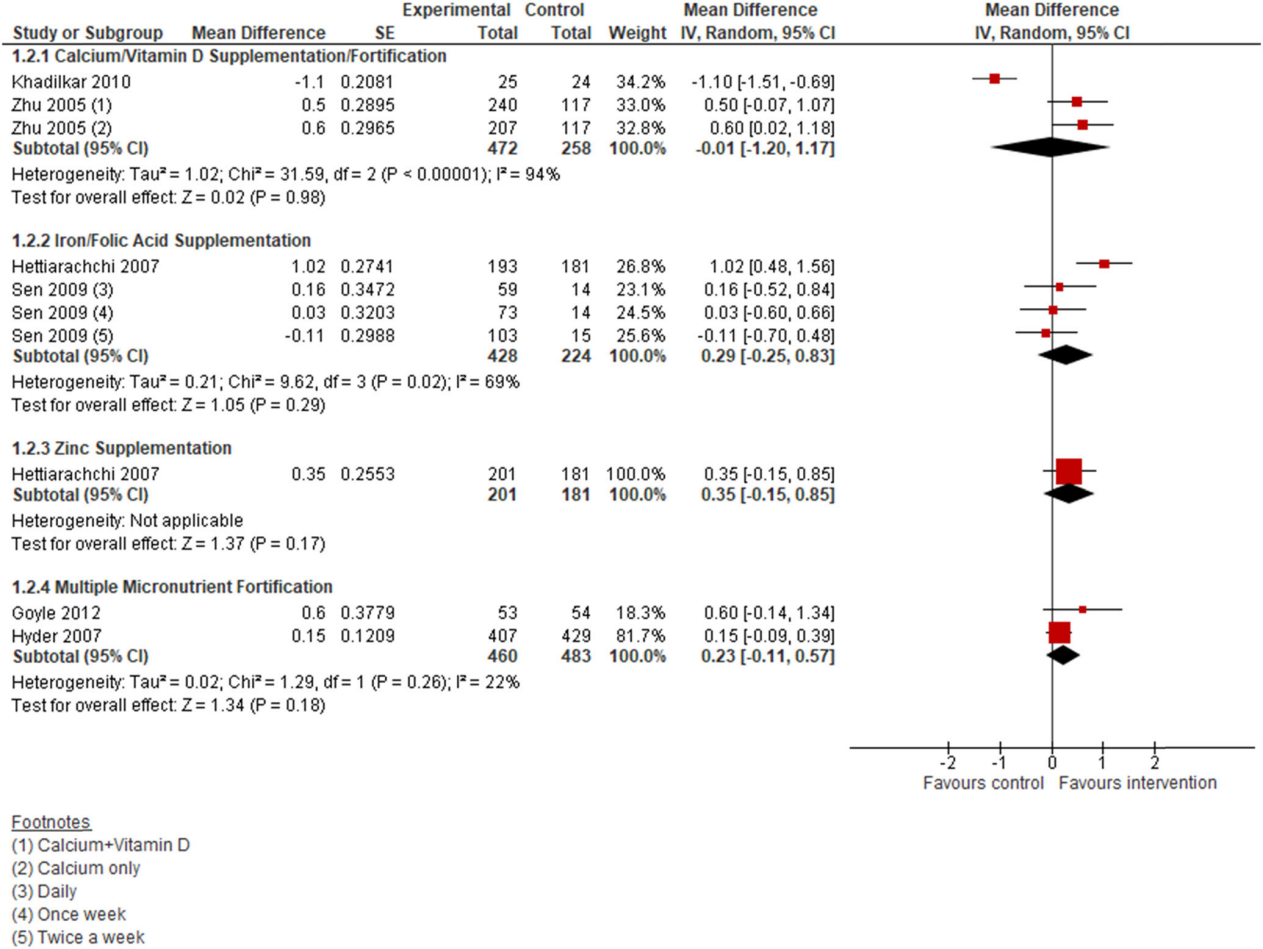
(Analysis 1.2) Forest plot of comparison: 1 Micronutrient Supplementation/Fortification versus No Supplementation/Fortificaton, outcome: 1.2 BMI

We are uncertain of the effect of the following micronutrient supplementation on BMI (Figure 5):

- Calcium/vitamin D supplementation (MD: −0.01 kg/m^2^; 95% CI: −1.20, 1.17; two studies; 730 participants; *I* ^2^ 94%; very low quality evidence; Analysis 1.2),
- Iron supplementation with or without folic acid (MD: 0.29 kg/m^2^; 95% CI: −0.25, 0.83; two studies; 652 participants; *I* ^2^ 69%; very low quality evidence; Analysis 1.2)
- Zinc supplementation (MD: 0.35 kg/m^2^; 95% CI: −0.15, 0.85; one study; 382 participants; very low quality evidence; Analysis 1.2)

MMN fortification (MD: 0.23 kg/m^2^, 95% CI: −0.11, 0.57; two studies; 943 participants; *I* ^2^ 22%; very low quality evidence; Analysis 1.2)

##### 5.3.2.2 Secondary outcomes

Among secondary outcomes, included studies reported haemoglobin concentrations, micronutrient status (zinc and vitamin D levels), body composition (total body BMC and total body BMD) and cognitive outcomes. None of the other secondary outcomes including other development outcomes and all‐cause mortality were reported.

###### 5.3.2.2.1 Haemoglobin concentrations: Pooled study result

**Figure 6:**
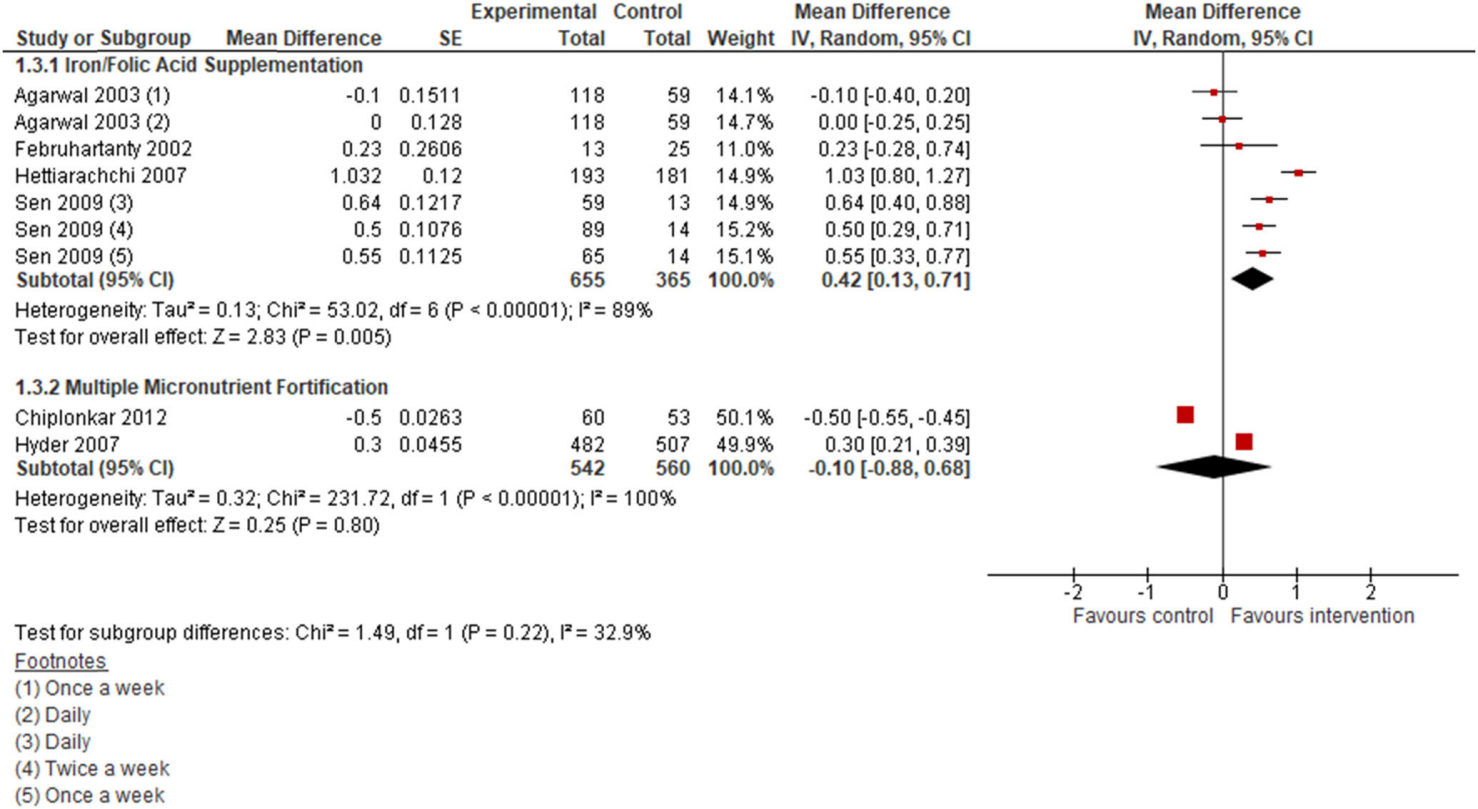
(Analysis 1.3) Forest plot of comparison: 1 Micronutrient Supplementation/Fortification versus No Supplementation/Fortificaton, outcome: 1.3 Haemoglobin

Iron supplementation with or without folic acid may improve haemoglobin concentrations among adolescents when compared to no supplementation (MD: 0.42 g/dL, 95% CI: 0.13, 0.71; four studies; 1020 participants; *I* ^2^ 89%; low quality evidence; Analysis 1.3; Figure 6). We are uncertain of the effect of MMN fortification on haemoglobin concentrations when compared to no fortification (MD: −0.10 g/dL, 95% CI: −0.88, 0.68; two studies; 1,102 participants; *I* ^2^ 100%; low quality evidence; Analysis 1.3; Figure 6).

Findings from Soekarjo et al. (2004) suggest that there was no significant difference in haemoglobin concentration with iron supplementation, vitamin A supplementation and iron + vitamin A supplementation compared to no supplementation among prepubertal or pubertal girls and boys.

###### 5.3.2.2.2 Micronutrient status: Pooled study result

Calcium/vitamin D supplementation may improve serum 25(OH) D levels (SMD: 2.85, 95% CI: 0.89, 4.82; two studies; 395 participants; *I* ^2^ 99%; low quality evidence; Analysis 1.4). We are uncertain of the effect of zinc supplementation on serum zinc levels (SMD: 6.94, 95% CI: −4.84, 18.71; two studies; 494 participants; *I* ^2^ 99%; low quality evidence; Analysis 1.5).

Findings from Soekarjo et al. (2004) suggest that vitamin A supplementation improved serum retinol concentration of boys, but not girls (0.33 in vitamin A supplementation group compared to 0.07 mmol/L in controls group).

###### 5.3.2.2.3 Body composition: Single study result

We are uncertain of the effect of calcium only supplementation (MD: 30.20 g, 95% CI: −40.56, 100.96; one study; 233 participants; low quality evidence; Analysis 1.6) and calcium + vitamin D supplementation (MD: 21.60 g, 95% CI: −45.32, 88.52; one study; 235 participants; low quality evidence; Analysis 1.6) on total body BMC.

We are uncertain of the effect of calcium only supplementation (MD: 0.02 g/cm^2^, 95% CI: −0.00, 0.04; one study; 233 participants; low quality evidence; Analysis 1.7) and calcium + vitamin D supplementation (MD: 0.02 g/cm^2^, 95% CI: −0.00, 0.04; one study; 235 participants; low quality evidence; Analysis 1.7) on total body BMD.

###### 5.3.2.2.4 Development outcomes: Single study result

**Figure 7:**
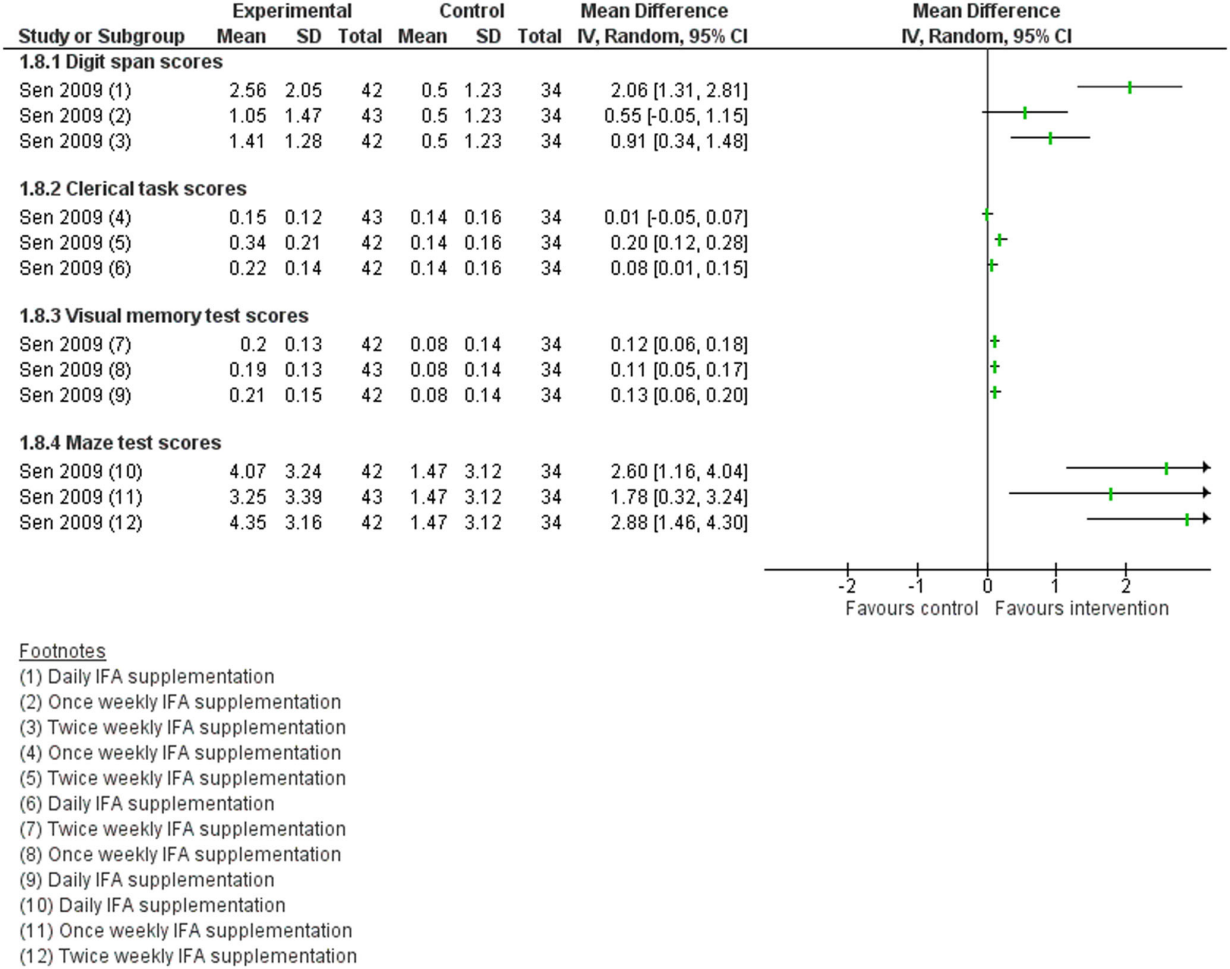
(Analysis 1.8) Forest plot of comparison: 1 Micronutrient Supplementation/Fortification versus No Supplementation/Fortificaton, outcome: 1.8 Cognitive outcomes

One study Sen 2009 reported the impact of iron supplementation with or without folic acid on cognition of adolescent girls suggesting improved digit span scores, clerical task scores, visual memory test scores and maze test scores in daily or twice weekly supplementation compared to once weekly or no supplementation (Analysis 1.8; Figure 7).

#### 5.3.3 Comparison 3: Macronutrients supplementation

We did not find any study assessing the impact of macronutrient supplementation on health and nutritional status among adolescents in LMICs.

## 6 DISCUSSION

### 6.1 Summary of main results

This review summarises findings from a total of 10 studies from 15 papers and including 10,802 participants. All the studies included in this review were RCTs and assessed the impact of micronutrient supplementation/fortification on health and nutritional status among adolescents in LMIC. We did not find any study assessing the impact of nutrition education and counselling or macronutrient supplementation. Micronutrient supplementation/fortification interventions included calcium/vitamin D supplementation/fortification; iron supplementation with or without folic acid; zinc supplementation; and MMN fortification. We did not find any large scale programmes evaluating nutrition interventions among adolescents in LMICs. We could not conduct any prespecified subgroup analysis due to limited number of studies.

In light of the WHO building blocks framework, the service delivery platform in all the included studies was school. The nutrition interventions were delivered through school teachers and student class monitors along with the study investigator. None of the included studies specified details pertaining to the health information system. In all of the included studies, the nutrition supplement was provided by the researcher while financing was provided by various not‐for‐profit organisations. In all of the included studies, study investigators led the intervention.

Among primary outcomes, we are uncertain of the effect of either daily or weekly supplementation of iron supplementation with or without folic acid on anaemia. We are also uncertain of the effect of calcium/vitamin D supplementation, iron supplementation with or without folic acid, zinc supplementation and MMN fortification on BMI among adolescents compared to no supplementation/fortification. None of the included studies reported any other primary outcome including morbidity or adverse effects.

Among secondary outcomes, included studies reported haemoglobin concentrations, micronutrient status (for serum zinc and serum vitamin D), body composition (total body BMC and total body BMD) and cognitive outcomes. Findings suggest that iron/folic acid supplementation may improve haemoglobin concentrations and calcium/vitamin D supplementation may improve serum 25(OH) D levels. We are uncertain of the effect of calcium only supplementation and calcium + vitamin D supplementation on total body BMD. We are uncertain of the effect of MMN fortification on haemoglobin concentrations; calcium supplementation on total body BMC, calcium + vitamin D supplementation on total body BMC and zinc supplementation on zinc levels. One study reported the impact of iron supplementation with or without folic acid on cognition of adolescent girls suggesting improved cognition in most of the tests with daily or twice weekly supplementation compared to once weekly or no supplementation. None of the other secondary outcomes including body composition, other development outcomes and all‐cause mortality were reported.

These findings warrant caution in interpretation due to the fact that there were very few studies and most had high heterogeneity and since they quality of the outcomes were either low or very low these can only be seen as preliminary findings. Moreover, we could not explore the possible causes of heterogeneity through subgroup or sensitivity analysis due to very few studies included in the review.

### 6.2 Overall completeness and applicability of evidence

This review summarises evidence on the effects of nutrition interventions among adolescents in LMICs. There were ten studies on micronutrient supplementation and fortification and all of the included studies except two targeted adolescent girls; two studies included both male and female adolescents. The duration of intervention varied from 10 week intervention, 4 months intervention, 6 months intervention, 1 year intervention to a maximum of 2 years of intervention. None of the included studies assessed the impact of nutrition education/counselling and macronutrient supplementation on health and nutrition outcomes among adolescents. The findings are generaliseable mainly for adolescent girls since all studies (except two) targeted female adolescents.

Since we did not find any large scale programmes assessing preventive nutrition interventions for adolescents in LMIC, we could not conduct an in‐depth analysis of the contextual factors that might potentially impact the effect of nutrition interventions in this age group in the light of the WHO building blocks. Almost all the included studies reported “service delivery”, “health workforce”, “access to essential medicines/supplies”, “financing” and “leadership/governance” while none of the included studies reported on “health information systems”. Findings from the included studies suggest that in LMICs, school based delivery of nutrition interventions remains the most utilised platform to target adolescents since the service delivery platform in all the included studies was school while the “health workforce” included school teachers and class monitors in majority of the included studies. The leadership and governance in almost all the studies remained under the researchers while financing was provided by various not‐for‐profit organisations. None of the included studies reported any information regarding “health information system”; since the data control ad monitoring was limited to the study period and were as per protocol and planned by the researcher. In all of the included studies, the nutrition supplement was provided by the researcher.

### 6.3 Quality of the evidence

Overall, the included studies were judged to be at unclear risk of bias due to insufficient information regarding sequence generation and allocation concealment. Majority of the included studies lacked blinding and were judged to be at high risk or unclear risk for blinding. Majority of the studies were at low risk of bias for incomplete outcome data, selective reporting and other biases.

The quality of the evidence was rated to be low to very low. The outcome quality was downgraded due to study limitations, including unclear sequence generation and allocation concealment methods and lack of blinding; high heterogeneity and imprecision.

### 6.4 Potential biases in the review process

The potential biases in the review process were that this type of review requires to make a number of subjective judgements and others may have reached different decisions regarding assessments of eligibility and risk of bias. We have tried to minimise these in two ways: (a) eligibility for inclusion and data extraction were assessed independently by two review authors and (b) assessments of risk of bias and data entry were also assessed independently by two review authors. We would encourage readers to examine the Characteristics of included studies tables to assist in the interpretation of results.

### 6.5 Agreements and disagreements with other studies or reviews

Two systematic reviews by Salam Rehana et al. (2016) and Lassi Zohra et al. (2017) assessed the effects of micronutrient supplementation. Both the reviews concluded that iron‐folic acid supplementation reduces anaemia; while our review findings are uncertain regarding any impact on anaemia with iron‐folic acid supplementation. The difference between these reviews and our review is that these reviews included youth (15–24 years of age) along with the adolescents while our review was restricted to the adolescent age group only. Many of the studies included in these reviews were excluded from our review due to the age cut‐offs. Therefore, the number of eligible studies in these reviews was greater than our review and our findings for anaemia is based on a single study. Futhermore, these reviews included studies from upper middle income and high income countries along with LMIC while our review only included studies conducted in LMICs.

The review by Das et al. (2013) assessed the impact of micronutrient fortification. This review concluded that MMN fortification significantly improved anaemia and haemoglobin concentrations; however this review also included overlapping age groups of children and adolescents and studies from upper middle income and high income countries.

There were very few studies in each comparison in our review and that could be the reason that we could not find any definite evidence on the outcomes.

## 7 AUTHORS' CONCLUSIONS

### 7.1 Implications for practice

The evidence on preventive nutrition interventions among adolescents from LMICs is too scarce for any conclusive implications for practice. The existing evidence is limited to micronutrient supplementation/fortification only while there is no evidence on nutrition education and counselling and macronutrient supplementation among adolescents.

### 7.2 Implications for research

Future studies assessing preventive nutrition interventions among adolescents should focus on assessing the effectiveness of nutrition education and macronutrient supplementation. There is a lack of focus on LMIC for this critical age group. Future studies should be well‐designed with appropriate follow‐up periods and also assessing any adverse effects. Large scale nutrition intervention programme evaluations are needed from LMIC settings. Future large scale nutrition programmes targeting adolescents in LMICs should also report the various contextual factors involved in planning, implementation and evaluation of these programmes in the light of the WHO health system building blocks. These data gaps are crucial for not only the sustainability of such programmes but also replication of the programmes in similar country settings. Existing studies have mainly targeted adolescent girls however future studies should target both adolescent boys and girls.

## AUTHOR CONTRIBUTIONS

All review authors (J. K. D., R. A. S., O. I., W. A., S. S. S. and Z. A. B.) contributed to the development of the review. R. A. S., J. K. D., O. I., W. A. and S. S. S. selected which studies to include, obtained copies of the studies and extracted data from the studies. O. I., W. A., S. S. S. and R. A. S. entered data into RevMan, carried out the analysis and interpreted the results. J. K. D., R. A. S., and Z. A. B. drafted the final review. As the contact author, Z. A. B. has overall responsibility for the review.

## CONFLICT OF INTERESTS

The authors declare that there are no conflict of interests.

## DIFFERENCES BETWEEN PROTOCOL AND REVIEW

We could not conduct any of the prespecified subgroup analysis and sensitivity analysis due to very few studies included in the each comparison in the review.

## PUBLISHED NOTES

**Characteristics of studies**

**Characteristics of included studies**

Agarwal et al. (2003)

**Table:** 

**Methods**	**Design**: RCT
	**Unit of Randomisation**: Cluster randomised trial. (Classes were clusters)
**Participants**	**Location/Setting**: Study was carried out at four Government Senior Secondary Schools, Delhi, India
	**Sample size**: 2088 adolescent girls
	**Dropouts/withdrawals**: 233 loss to follow‐up out of 2,088
	**Sex**: Only girls
	**Mean age**: Not specified
	**Inclusion criteria**: Girls aged 10–17 years
	**Exclusion criteria**: Girls with haemoglobin <7.0 g/dL were excluded
**Interventions**	**Intervention (sample size)**:
	100 mg elemental iron and 500 micrograms folic acid in the form of oral tablets was provided for 100 days
	Group 1: Daily treatment (*N* = 702)
	Group 2: Weekly treatment: (*N* = 695)
	**Control (sample size)**:
	Control group did not receive any tablets during the intervention period and haemoglobin was estimated at 115 ± 5 days. They were thereafter given 100 tablets with advice to take 1 tablet daily for 100 days (*N* = 691)
**Outcomes**	**Primary outcomes**: Hemoglobin, plasma ferritin, anaemia
	**Secondary outcomes**: Not specified
	**Timing of outcome assessment**: 115 days and 230 days
**Notes**	**Study start date**: August 1996
	**Study end date**: Februray 1999
	**Funding source**: UNICEF, New Delhi
	**Conflicts of interest**: None stated

**Risk of bias table**

**Table:** 

Bias	Authors' judgement	Support for judgement
Random sequence generation (selection bias)	Unclear risk	Quote: “As school teachers did not agree to randomisation at the individual girl level, the randomisation was done at the class section level for the 60 class sections (all class sections taken).”
		Comment: Insufficient information to permit judgement
Allocation concealment (selection bias)	Unclear risk	Comment: Insufficient information to permit judgement
Blinding of participants and personnel (performance bias)	High risk	Comment: Probably not done
Blinding of outcome assessment (detection bias)	Unclear risk	Comment: Insufficient information to permit judgement
Incomplete outcome data (attrition bias)	Low risk	Comment:
		Group 1: 121/702 loss to follow‐up
		Group 2: 0/695 loss to follow‐up
		Group 3: 112/691 loss to follow‐up
Selective reporting (reporting bias)	Unclear risk	Comment: Trial registration not reported. Outcomes specified in the methodology section were reported.
Other bias	Low risk	Comment: Sample size assumptions are not specified.

Chiplonkar and Kawade (2012)

**Table:** 

**Methods**	**Design**: RCT
	**Unit of randomisation**: Individually randomised trial
**Participants**	**Location/Setting**: A secondary girls school in Pune City, Maharashtra, India
	**Sample size**: 180 apparently healthy adolescent girls
	**Dropouts/withdrawals**: 8 loss to follow‐up of 180
	**Sex**: Adolescent girls only
	**Mean age**: 12.5 (0.85) years
	**Inclusion criteria**: Apaarently healthy adolescent girls
	**Exclusion criteria**: Girls who were ill or had in the recent past any illness such as fever, respiratory or gastrointestinal infection, or those undergoing medical treatment, or taking multivitamin mineral supplements were identified and excluded from the study
**Interventions**	**Intervention (sample size)**:
	Intervention group 1 (*N* = 60)
	Supplement was provided in the form of six different snacks to each girl with one snack (average amount 100 g/serving) per day for 6 school days in a week. The average zinc content of the food supplements was 2.2 ± 0.4 mg/serving
	Intervention group 2 (*N* = 59)
	The ayurvedic zinc tablet containing 20 mg of jasad bhasma, equivalent to 16.6 mg of elemental zinc, was given to each girl every day for 6 school days/week under the guidance of an ayurvedic doctor
	The intervention was provided for a duration of 10 weeks
	**Control (sample size)**:
	Control group (*N* = 53)
	No supplements given to control
**Outcomes**	**Primary outcomes**: Dietry intake, haemoglobin levels, plasma zinc, plasma beta‐carotene, plasma retinol, plasma vitamin C
	**Secondary outcomes**: Not specified
	**Timing of outcome assessment**: After 10 weeks of intervention
**Notes**	**Study start date**: Not specified
	**Study end date**: Not specified
	**Funding source**: Zensar Foundation, Pune, India
	**Conflicts of interest**: Not specified

**Risk of bias table**

**Table:** 

Bias	Authors' judgement	Support for judgement
Random sequence generation (selection bias)	Unclear risk	Comment: Insufficient information to permit judgement
Allocation concealment (selection bias)	Unclear risk	Comment: Insufficient information to permit judgement
Blinding of participants and personnel (performance bias)	High risk	Comment: Probably not done
Blinding of outcome assessment (detection bias)	Unclear risk	Comment: Insufficient information to permit judgement
Incomplete outcome data (attrition bias)	Low risk	Comment:
		Group 1: No loss to follow up
		Group 2: 1/60 loss to follow‐up
		Group 3: 7/60 loss to follow‐up
Selective reporting (reporting bias)	Unclear risk	Comment: Trial registration not reported. Outcomes specified in the methodology section were reported
Other bias	Low risk	Comment: No other biases detected.

Februhartanty et al. (2002)

**Table:** 

**Methods**	**Design**: RCT
	**Unit of randomisation**: Individually randomised trial
**Participants**	**Location/Setting**: Junior high schools in Kupang, East Nusa Tenggara, in the eastern part of Indonesia
	**Sample size**: 150 female adolescents
	**Dropouts/withdrawals**: 13 out of 150 loss to follow‐up
	**Sex**: Only female adolescents
	**Mean age**: 14.6 (1.1) years
	**Inclusion criteria**: Postmenarcheal female adolescent
	**Exclusion criteria**: Not specified
**Interventions**	**Intervention (sample size)**:
	The iron tablet used in this study contained 60 mg elemental iron and 0.25 mg folic acid in the form of 200 mg ferrous sulphate
	Group 1: Weekly iron tablets (*N* = 50)
	Group 2: Iron tablet for four consecutive days during their menstruation cycle (*N* = 50)
	**Control (sample size)**:
	Placebo tablet (*N* = 50)
	The supplementation was conducted over 16 weeks under the supervision of teachers appointed from the participating schools and the first author. To control parasitic infestation, all subjects were given a single dose of 500 mg mebendazole three days before supplementation
**Outcomes**	**Primary outcomes**: Hemoglobin level, ferritin level
	**Secondary outcomes**: Not specified
	**Timing of outcome assessment**: After 16 weeks of intervention
**Notes**	**Study start date**: August 1998
	**Study end date**: December 1998
	**Funding source**: SEAMEO‐TROPMED Regional Center for Community Nutrition in Jakarta
	**Conflicts of interest**: Not specified

**Risk of bias table**

**Table:** 

Bias	Authors' judgement	Support for judgement
Random sequence generation (selection bias)	Unclear risk	Quote: “.and allocated randomly to placebo or weekly groups.”
		Comment: Insufficient information to permit judgement
Allocation concealment (selection bias)	Unclear risk	Quote: “One hundred of them were recruited from one school and allocated randomly to placebo or weekly groups. The other 50 students were recruited at random from a different junior high school and allocated to the menstruation group.”
		Comment: Insufficient information to permit judgement
Blinding of participants and personnel (performance bias)	Low risk	Quote: “This single blind community experimental study.”
		Comment: Adequately done
Blinding of outcome assessment (detection bias)	High risk	Quote: “This single blind community experimental study.”
		Comment: Not done
Incomplete outcome data (attrition bias)	Low risk	Comment:
		Group 1: 2/50 loss to follow‐up
		Group 2: 9/50 loss to follow‐up
		Group 3: 2/50 loss to follow‐up
Selective reporting (reporting bias)	Unclear risk	Comment: Trial registration not reported. Outcomes specified in the methodology section were reported
Other bias	Low risk	Comment: No other biases detected

Goyle (2012)

**Table:** 

**Methods**	**Design**: RCT
	**Unit of randomisation**: Individually randomised trial
**Participants**	**Location/Setting**: Government school near university of Rajasthan,Jaipur, India
	**Sample size**: 107 adolescent girls
	**Dropouts/withdrawals**: No loss to follow‐up
	**Sex**: Only female adolescents
	**Mean age**: Not specified
	**Inclusion criteria**: All adolescent girls studying in classes VI to VIII were enrolled
	**Exclusion criteria**: Not specified
**Interventions**	**Intervention (sample size)**:
	Intervention group (*N* = 53): 100 g of biscuits fortified with one RDA levels of vitamin A, iron, folic acid, vitamin C and iodine was provided for all working days during 4 months (total 75 days of supplementation)
	**Control (sample size)**:
	Placebo (*N* = 54): 100 g of biscuits furnishing 497 kcal and 11.36 g of protein per day were provided to the control group for 4 months
**Outcomes**	**Primary outcomes**: BMI, BMI z‐scores, weight‐for‐age, height‐for‐age
	**Secondary outcomes**: Not specified
	**Timing of outcome assessment**: After 4 months of intervention
**Notes**	**Study start date**: September 2004
	**Study end date**: December 2004
	**Funding source**: University Grants Commission, New Delhi, India
	**Conflicts of interest**: Not specified

**Risk of bias table**

**Table:** 

Bias	Authors' judgement	Support for judgement
Random sequence generation (selection bias)	Unclear risk	Quote: “The girls were randomly allocated to the control and experimental groups.”
		Comment: Insufficient information to permit judgement
Allocation concealment (selection bias)	Unclear risk	Quote: “The girls were randomly allocated to the control and experimental groups.”
		Comment: Insufficient information to permit judgement
Blinding of participants and personnel (performance bias)	High risk	Comment: Probably not done
Blinding of outcome assessment (detection bias)	High risk	Comment: Probably not done
Incomplete outcome data (attrition bias)	Low risk	Comment:
		Group 1: 0/53 loss to follow‐up
		Group 2: 0/53 loss to follow‐up
Selective reporting (reporting bias)	Unclear risk	Comment: Trial registration not reported. Outcomes specified in the methodology section were reported
Other bias	Low risk	Comment: Sample size assumptions were not specified

Hettiarachchi et al. (2008)

**Table:** 

**Methods**	**Design**: RCT
	**Unit of randomisation**: Individually randomised trial
**Participants**	**Location/Setting**: School in the Galle district, Sri Lanka
	**Sample size**: 821 school children
	**Dropouts/withdrawals**: 47 out of 821 loss to follow‐up
	**Sex**: Both male and female children were enrolled
	**Mean age**: 13.5 years
	**Inclusion criteria**: Children with Hb level >80 g/L were eligible for the study
	**Exclusion criteria**: Children suffering from acute or chronic diseases, inflammatory conditions, giving a history of any drug consumption other than paracetamol or antihistamines for minor ailments, currently consuming nutrient supplements or having donated blood or received a blood transfusion within the last 4 months were excluded from the study
**Interventions**	**Intervention (sample size)**:
	Children were supplemented with two capsules per day containing either:
	Group 1: Iron (50 mg/day) in the form of ferrous fumarate (*N* = 202)
	Group 2: Zinc (14 mg/day) in the form of zinc sulphate (*N* = 213)
	Group 3: Combined (iron + zinc) (*N* = 216)
	**Control (sample size)**:
	Group 4: Placebo made of anhydrous lactose (*N* = 190)
	Intervention was provided on school days for a duration of 24 weeks
**Outcomes**	**Primary outcomes**: Height, weight, BMI, height‐for‐age, weight‐for‐age, stunted, underweight, haemoglobin, serum zinc, serum ferritin
	**Secondary outcomes**: Not specified
	**Timing of outcome assessment**: After 24 weeks of intervention
**Notes**	**Study start date**: Not specified
	**Study end date**: Not specified
	**Funding source**: International Atomic Energy Agency (IAEA‐SRL‐11958)
	**Conflicts of interest**: Not specified

**Risk of bias table**

**Table:** 

Bias	Authors' judgement	Support for judgement
Random sequence generation (selection bias)	Unclear risk	Quote: “Subjects were randomised into one of four groups where randomization was stratified by classroom using a double‐blind approach”
		Comment: Insufficient information to permit judgement
Allocation concealment (selection bias)	Unclear risk	Quote: “Subjects were randomised into one of four groups where randomizations was stratified by classroom using a double‐blind approach”
		Comment: Insufficient information to permit judgement
Blinding of participants and personnel (performance bias)	Low risk	Quote: “Subjects were randomised into one of four groups where randomizations was stratified by classroom using a double‐blind approach.”
		Comment: Adequately done
Blinding of outcome assessment (detection bias)	Unclear risk	Quote: “Subjects were randomised into one of four groups where randomizations was stratified by classroom using a double‐blind approach”
		Comment: Insufficient information to permit judgement
Incomplete outcome data (attrition bias)	Low risk	Comment:
		Group 1: 9/202 loss to follow‐up
		Group 2: 12/213 loss to follow‐up
		Group 3: 17/216 loss to follow‐up
		Group 4: 9/190 loss to follow‐up
Selective reporting (reporting bias)	Unclear risk	Comment: Trial registration not reported. Outcomes specified in the methodology section were reported
Other bias	Low risk	Comment: No other biases detected

Hyder et al. (2007)

**Table:** 

**Methods**	**Design**: RCT
	**Unit of randomisation**: Individually randomised trial
**Participants**	**Location/Setting**: Conducted in 54 nonformal primary education schools operated by the Bangladesh Rural Advancement Committee (BRAC, one of the largest national nongovernmental organizations in the world) in Sherpur district, 300 km northeast of Dhaka city
	**Sample size**: 1125 adolescent girls
	**Dropouts/withdrawals**: 136 out of 1,125 loss to follow‐up
	**Sex**: Only adolescent girls
	**Mean age**: 12 years
	**Inclusion criteria**: Not specified
	**Exclusion criteria**: Students suffering from either severe micronutrient deficiencies or acute infection (clinical signs of fever or reportedly suffer from any infectious disease) were excluded and referred to the nearby health center for appropriate treatment
**Interventions**	**Intervention (sample size)**:
	Group 1 (*N* = 559): Powdered beverage fortified with multiple‐micronutrients and packaged in sachets
	**Control (sample size)**:
	Group 2 (*N* = 566): Placebo beverage
	The test beverages were consumed 6 days per week for 12 months at the schools
**Outcomes**	**Primary outcomes**: Weight, height, MUAC, BMI, haemoglobin, serum ferritin, serum retinol, serum zinc
	**Secondary outcomes**: Not specified
	**Timing of outcome assessment**: After 12 months of intervention
**Notes**	**Study start date**: Not specified
	**Study end date**: Not specified
	**Funding source**: Micronutrient Initiative, Ottawa, Canada
	**Conflicts of interest**: No conflicts of interest declared

**Risk of bias table**

**Table:** 

Bias	Authors' judgement	Support for judgement
Random sequence generation (selection bias)	Low risk	Quote: “Randomization was done by listing all selected children, assigning them with random numbers, and dividing the odd numbers from the even numbers to form the 2 groups.”
		Comment: Adequately done
Allocation concealment (selection bias)	Low risk	Quote: “Randomization was done by listing all selected children, assigning them with random numbers, and dividing the odd numbers from the even numbers to form the 2 groups.”
		Comment: Adequately done
Blinding of participants and personnel (performance bias)	Low risk	Quote: “One shastho shebika was assigned per school to prepare and distribute the drink. Students did not know whether the blue or yellow Coloured sachets contained the fortified beverage”
		Comment: Adequately done
Blinding of outcome assessment (detection bias)	Low risk	Quote: “Researchers, schoolteachers, shasthoshebikas, and students did not know whether the blue or yellow Coloured
		sachets contained the fortified beverage.” “The decoding was done only by the manufacturer after the study was completed and the data analysed.”
		Comment: Adequately done
Incomplete outcome data (attrition bias)	Low risk	Comment:
		Group 1: 77/559
		Grup 2: 59/566
Selective reporting (reporting bias)	Unclear risk	Comment: Trial registration not reported. Outcomes specified in the methodology section were reported
Other bias	Low risk	Comment: No other biases detected.

Khadilkar et al. (2010)

**Table:** 

**Methods**	**Design**: RCT
	**Unit of randomisation**: Individually randomised trial
**Participants**	**Location/Setting**: State run school in Pune, India
	**Sample size**: 50 adolescent girls
	**Dropouts/withdrawals**: 1 out of 50 loss to follow‐up
	**Sex**: Only adolescent post menarchal girls
	**Mean age**: 14.6 years
	**Inclusion criteria**: Post Menarche adolescent girls
	**Exclusion criteria**: Not specified
**Interventions**	**Intervention (sample size)**:
	Group 1 (*N* = 25): Subjects in the treatment group were administered 6 vitamin D2 (Ergocalciferol; Celltech, UK) tablets each containing 1.25 mg
	(50,000 IU) orally at 1, 4, 7 and 10 months
	**Control (sample size)**:
	Group 2 (*N* = 25): Placebo group the local pharmacist prepared tablets which were identical in number, colour, size and texture to the ergocalciferol, but contained only sucrose
	The intervention was provided for a duration of one year and all participants received 250 mg elemental calcium (calcium carbonate) daily
**Outcomes**	**Primary outcomes**: Total body bone mineral content, Lumbar spine bone mineral content and lumbar spine bone mineral apparent density
	**Secondary outcomes**: Total body lean, fat mass and serum concentrations of biochemical parameters
	**Timing of outcome assessment**: After one year of intervention
**Notes**	**Study start date**: February 2006
	**Study end date**: April 2007
	**Funding source**: Not specified
	**Conflicts of interest**: None declared

**Risk of bias table**

**Table:** 

Bias	Authors' judgement	Support for judgement
Random sequence generation (selection bias)	Unclear risk	Quote: “.participants were randomised by the trial statistician (MS) into two groups”
		Comment: Insufficient information to permit judgement
Allocation concealment (selection bias)	Unclear risk	Quote: “.participants were randomised by the trial statistician (MS) into two groups”
		Comment: Insufficient information to permit judgement
Blinding of participants and personnel (performance bias)	Low risk	Quote: “local pharmacist prepared tablets which were identical in number, colour, size and texture to the ergocalciferol, but contained only sucrose.”
		Comment: Adequately done
Blinding of outcome assessment (detection bias)	Low risk	Quote: “All the primary investigators of the study were totally blinded to the treatment regimen.”
		Comment: Adequately done.
Incomplete outcome data (attrition bias)	Low risk	Comment:
		Group 1: No loss to follow‐up.
		Group 2: 1/50 loss to follow‐up.
Selective reporting (reporting bias)	Unclear risk	Comment: Trial registration not reported. Outcomes specified in the methodology section were reported
Other bias	Low risk	Comment: No other biases detected.

**Sen** (2009)

**Table:** 

**Methods**	**Design**: RCT
	**Unit of randomisation**: Cluster (school) randomised trial
**Participants**	**Location/Setting**: Municipal primary schools in Vadodara, India
	**Sample size**: 358 girls
	**Dropouts/withdrawals**: 104 out of 358 loss to follow‐up
	**Sex**: Only girls
	**Mean age**: Not specified
	**Inclusion criteria**: 9–13 year old girls studying in grades V and VI were enrolled in the study
	**Exclusion criteria**: Not specified
**Interventions**	**Intervention (sample size)**:
	Group 1 (*N* = 94): The participants were given IFA tablets (100 mg elemental iron + 0.5 mg folic acid) once weekly
	Group 2 (*N* = 118): The participants were given IFA tablets (100 mg elemental iron + 0.5 mg folic acid) twice weekly
	Group 3 (*N* = 81): The participants were given IFA tablets (100 mg elemental iron + 0.5 mg folic acid) daily
	The intervention was continued for a duration of 1 year
	**Control (sample size)**:
	Group 4 (*N* = 65): Control group did not receive any intervention
**Outcomes**	**Primary outcomes**: Hemoglobin levels, BMI, cognitive test scores
	**Secondary outcomes**: Not specified
	**Timing of outcome assessment**: After one year of intervention
**Notes**	**Study start date**: Not specified
	**Study end date**: Not specified
	**Funding source**: None
	**Conflicts of interest**: None stated

**Risk of bias table**

**Table:** 

Bias	Authors' judgement	Support for judgement
Random sequence generation (selection bias)	Unclear risk	Quote: “.four schools were randomly sampled from the sampling universe of 17 schools”
		Comment: Insufficient information to permit judgement
Allocation concealment (selection bias)	Unclear risk	Quote: “.four schools were randomly sampled from the sampling universe of 17 schools”
		Comment: Insufficient information to permit judgement
Blinding of participants and personnel (performance bias)	High risk	Quote: “The investigators maintained regular supply of IFA, supervised the distribution and recorded compliance in all the schools.”
		Comment: not adequately done
Blinding of outcome assessment (detection bias)	High risk	Quote: “The investigators maintained regular supply of IFA, supervised the distribution and recorded compliance in all the schools.”
		Comment: not adequately done
Incomplete outcome data (attrition bias)	High risk	Comment:
		Group 1: 29/94
		Group 2: 29/118
		Group 3: 22/81
		Group 4: 24/65
Selective reporting (reporting bias)	Unclear risk	Comment: Trial registration not reported. Outcomes specified in the methodology section were reported
Other bias	Low risk	Comment: No other biases detected.

Soekarjo et al. (2004)

**Table:** 

**Methods**	**Design**: RCT
	**Unit of randomisation**: cluster (school grade) randomised trial
**Participants**	**Location/Setting**: 24 schools in Indonesia from both urban and rural locations
	**Sample size**: 5,166 adolescents aged 12–15 years
	**Dropouts/withdrawals**: 356 out of 5,166 loss to follow‐up
	**Sex**: Both male and female adolescents were enrolled
	**Mean age**: 14.2 years
	**Inclusion criteria**: Sample was selected randomly from all adolescent pupils studying in the 24 schools selected
	**Exclusion criteria**: Not specified
**Interventions**	**Interventions (sample size)**:
	Group 1 (*N* = 1,033): weekly 10,000 IU vitamin A
	Group 2 (*N* = 1,045): weekly 60 mg elemental iron (as ferrous sulphate) plus 250 mg folate
	Group 3 (*N* = 1,130): weekly 10,000 IU vitamin A and 60 mg elemental iron plus 250 mg folate
	The supplements were given once weekly for 3 months (a total of 14 times).
	**Control (sample size)**:
	Group 4 (*N* = 1958): Did not receive any supplement
**Outcomes**	**Primary outcomes**: Haemoglobin concentration, Serum retinol concentrations
	**Secondary outcomes**: Not specified
	**Timing of outcome assessment**: After 3 months of intervention
**Notes**	**Study start date**: October 1996
	**Study end date**: May 1997
	**Funding source**: USAID through the OMNI project
	**Conflicts of interest**: Not specified

**Risk of bias table**

**Table:** 

Bias	Authors' judgement	Support for judgement
Random sequence generation (selection bias)	Unclear risk	Quote: “Adolescents from 15 schools (four U‐MTs, seven U‐SMP and four R‐SMP) (*n* = 2990) were randomly selected to receive weekly supplements, while adolescents in the other nine schools (three U‐MTs, two U‐SMP and four R‐SMP) served as controls (*n* = 1750).”
		Comment: Insufficient information to permit judgement.
Allocation concealment (selection bias)	Unclear risk	Quote: “In each of the schools receiving supplements, each of the three grades was randomly allocated to receive one of the three supplementation regimes.”
		Comment: Insufficient information to permit judgement.
Blinding of participants and personnel (performance bias)	High risk	Quote: “All pupils were aware of which supplement they were taking and they were told that the supplements would improve their health and prevent/cure anaemia”
		Comment: Not done
Blinding of outcome assessment (detection bias)	High risk	Quote: “All pupils were aware of which supplement they were taking and they were told that the supplements would improve their health and prevent/cure anaemia”
		Comment: Not done
Incomplete outcome data (attrition bias)	Low risk	Comment:
		Group 1: 63/1,033
		Group 2: 67/1,045
		Group 3: 88/1,130
		Group 4: 138/1,958 (data presented for control group on a stratified random sample of 626)
Selective reporting (reporting bias)	Unclear risk	Comment: Trial registration not reported. Outcomes specified in the methodology section were reported
Other bias	Low risk	Comment: No other biases detected

Zhu et al. (2005)

**Table:** 

**Methods**	**Design**: RCT
	**Unit of randomisation**: Indivdually randomised trial
**Participants**	**Location/Setting**: Schools in urban Beijing, China
	**Sample size**: 757 adolescent girls
	**Dropouts/withdrawals**: 59/757 loss to follow‐up
	**Sex**: Only adolescent girls
	**Mean age**: 10 (0.03) years
	**Inclusion criteria**: Healthy girls aged 10 years
	**Exclusion criteria**: Not specified
**Interventions**	**Intervention (sample size)**:
	Group 1 (*N* = 238): Girls consumed a carton of 330 ml milk fortified with Ca on school days over the study period
	Group 2 (*N* = 260): Girls received the same quantity of milk additionally fortified with 5 or 8 mg cholecalciferol
	The duration of intervention was 24 months
	**Control (sample size)**:
	Group 3 (*N* = 259): Control girls did not receive any intervention
**Outcomes**	**Primary outcomes**: Nutrient intake, bone mineral content, bone mineral density, serum PTH, serum calcium, height, weight and vitamin D levels
	**Secondary outcomes**: Not specified
	**Timing of outcome assessment**: Immediately after the 24 months of intervention and 3 years post intervention
**Notes**	**Study start date**: April 1999
	**Study end date**: March 2001
	**Funding source**: Australian Dairy Research and Development Corporation, Murray Goulburn Co‐operative Co. Limited (formulated and produced the milk supplements) and the Nestle' Foundation provided financial support for the laboratory analyses
	**Conflicts of interest**: None declared

**Risk of bias table**

**Table:** 

Bias	Authors' judgement	Support for judgement
Random sequence generation (selection bias)	Unclear risk	Quote: “The 9 schools were randomly assigned to 3 study groups”
		Comment: Insufficient information to permit judgement
Allocation concealment (selection bias)	Unclear risk	Quote: “The 9 schools were randomly assigned to 3 study groups”
		Comment: Insufficient information to permit judgement
Blinding of participants and personnel (performance bias)	Low risk	Quote: “Each milk supplement was supplied in colour‐coded UHT cartons with the identity of the supplement being unknown to both subjects and investigators during the course of the study.”
		Comment: Adequately done
Blinding of outcome assessment (detection bias)	Low risk	Quote: “Each milk supplement was supplied in colour‐coded UHT cartons with the identity of the supplement being unknown to both subjects and investigators during the course of the study.”
		Comment: Adequately done
Incomplete outcome data (attrition bias)	Low risk	Comment:
		Group 1:29/238 loss to follow‐up
		Group 2: 18/260 loss to follow‐up
		Group 3: 12/259 loss to follow‐up
Selective reporting (reporting bias)	Unclear risk	Comment: Trial registration not reported. Outcomes specified in the methodology section were reported
Other bias	Low risk	Comment: No other biases detected

**Characteristics of excluded studies**

**Table:** 

Abrams et al. (2005)	
**Reason for exclusion**	Intervention given was prebiotic (inulin‐type fructans)
Ahmed et al. (2005)	
**Reason for exclusion**	Participants were anaemic at baseline and the intervention was therapeutic
Ahmed et al. (2010)	
**Reason for exclusion**	Participants were anaemic at baseline and the intervention was therapeutic
Angeles‐Agdeppa et al. (1997)	
**Reason for exclusion**	Participants were asymptomatic anaemic individuals. Intervention was used as therapeutic intervention
Beasley et al. (2000)	
**Reason for exclusion**	Participants were infected with schistosomiasis. Infection is believed to affect the outcome. IFA taken as therapeutic intervention
Castillo‐Durán et al. (2001)	
**Reason for exclusion**	The study was from non‐LMIC country
Chan et al. (2006)	
**Reason for exclusion**	The study was carried out in a non LMIC country
Damsgaard et al. (2012)	
**Reason for exclusion**	The study included overweight individuals and was also conducted in a non‐LMIC country
**De Oliveiera 2009**	
**Reason for exclusion**	The study was from non‐LMIC country
Deshmukh et al. (2008)	
**Reason for exclusion**	This study did not have an appropriate control group
Diogenes et al. (2013)	
**Reason for exclusion**	The study was from non‐LMIC country
Dongre et al. (2011)	
**Reason for exclusion**	This study did not have an appropriate control group
Eftekhari et al. (2006)	
**Reason for exclusion**	Participants were iron deficient at baseline and the intervention was therapeutic
Friis et al. (1997)	
**Reason for exclusion**	93% of the Participants were infected by schistosomiasis. Infection is believed to affect the outcome
Ganmaa et al. (2017)	
**Reason for exclusion**	Participants were asymptomatic vitamin D deficient individuals according to the inclusion criteria. Intervention was used as therapeutic intervention
Ilich‐Ernst et al. (1998)	
**Reason for exclusion**	The study was carried out in a non LMIC Country
Kianfar et al. (2000)	
**Reason for exclusion**	The intervention was therapeutic
Kotecha et al. (2009)	
**Reason for exclusion**	The study does not have an appropriate control group
Lambert et al. (2008)	
**Reason for exclusion**	The study was carried out in a non LMIC Country
Ma et al. (2014)	
**Reason for exclusion**	The study does not have an appropriate control group
Manger et al. (2008)	
**Reason for exclusion**	The study population included children and adolescents and the study author suggested that the data for the adolescent subgroup was too small
Mann et al. (2002)	
**Reason for exclusion**	Participants were asymptomatic anaemic individuals. Grouping was done based on energy intakes
McKenna et al. (1997)	
**Reason for exclusion**	The study was carried out in a non LMIC Country
Mwaniki et al. (2002)	
**Reason for exclusion**	The intervention was therapeutic
Pilz et al. (2017)	
**Reason for exclusion**	The methods describe inclusion criteria of age 18–45 years but results show age of participants was between 22 and 29 years. Participants are not adolescents
Prentice et al. (2005)	
**Reason for exclusion**	The study was carried out in a non LMIC Country
Prentice et al. (2012)	
**Reason for exclusion**	The study population included children and adolescents. The corresponding authors were contacted for the adolescent subgroup data; however we did not receive any response
Rerksuppaphol and Rerksuppaphol (2016)	
**Reason for exclusion**	The study population included children and adolescents. The corresponding authors were contacted for the adolescent subgroup data; however we did not receive any response
Rousham et al. (2013)	
**Reason for exclusion**	Intervention was used as therapeutic intervention
Sarma et al. 2006)	
**Reason for exclusion**	The study population included children and adolescents. The corresponding authors were contacted for the adolescent subgroup data; however we did not receive any response
Schou et al. (2003)	
**Reason for exclusion**	The study was carried out in a non LMIC Country
Shah and Gupta (2002)	
**Reason for exclusion**	Intervention was used as therapeutic intervention
Silk et al. 2015)	
**Reason for exclusion**	The study was carried out in a non LMIC Country
Sunawang et al. (2009)	
**Reason for exclusion**	The participants were not adolescents
Tee et al. (1999)	
**Reason for exclusion**	There is no appropriate control group
Viljakainen et al. (2006)	
**Reason for exclusion**	The study was carried out in a non LMIC Country
White et al. (2015)	
**Reason for exclusion**	The study was carried out in a non LMIC Country
Yusoff et al. (2012)	
**Reason for exclusion**	The study was from non‐LMIC country

1. Summary of findings

**Table:** 

Micronutrient supplementation/fortification compared with placebo/no supplementation/fortification for health and nutritional status						
Patient or population: Adolescents						
Settings: School settings						
Intervention: Micronutrient supplementation/fortification						
Comparison: Placebo/no supplementation/fortification						
	Illustrative comparative risks* (95% CI)					
	Assumed risk	Corresponding risk				
Outcomes	Placebo/No supplementation/fortification	Micronutrient supplementation/fortification	Relative effect (95% CI)	No of Participants (studies)	Quality of the evidence (GRADE)	Comments
Daily Iron supplementation with or without folic acid: **Anaemia**	206 of 579	216 of 581	RR: 1.06 [0.95, 1.18]	1,160 participants (one study)	⊕⊕⊝⊝	
					**Low** ^a^, ^b^	
Weekly Iron supplementation with or without folic acid: **Anaemia**	206 of 579	265 of 695	RR: 1.07 [0.93, 1.24]	1,274 participants (one study)	⊕⊕⊝⊝	
					**Low** ^a^, ^b^	
Calcium/vitamin D supplementation/fortification: **BMI**	The mean BMI in the control group ranged between 18.15 and 18.5	The mean BMI in the intervention group ranged between 17.05 and 19.1	MD: −0.01 kg/m^2^ [−1.20, 1.17]	730 participants (two studies)	⊕⊝⊝⊝	
					**Very Low** ^a^, ^b^, ^c^	
Iron supplementation with or without folic acid: **BMI**	The mean BMI in the control group ranged between 15.78 and 16.23	The mean BMI in the intervention group ranged between 15.67 and 17.25	MD: 0.29 kg/m^2^ [−0.25, 0.83]	652 participants (two studies)	⊕⊝⊝⊝	
					**Very Low** ^a^, ^b^, ^c^	
Zinc supplementation: **BMI**	The mean BMI in the control group ranged was 16.23	The mean BMI in the intervention group was 16.58	MD: 0.35 kg/m^2^ [−0.15, 0.85]	382 participants (one study)	⊕⊝⊝⊝	
					**Very Low** ^a^, ^b^, ^c^	
MMN fortification: **BMI**	The mean BMI in the control group ranged between 15.27 and 16.5	The mean BMI in the intervention group ranged between 15.42 and 17.1	MD: 0.23 kg/m^2^ [−0.11, 0.57]	943 participants (two studies)	⊕⊝⊝⊝	
					**Very Low** ^a^, ^b^, ^c^	

GRADE Working Group grades of evidence: High quality: Further research is very unlikely to change our confidence in the estimate of effect. Moderate quality: Further research is likely to have an important impact on our confidence in the estimate of effect and may change the estimate. Low quality: Further research is very likely to have an important impact on our confidence in the estimate of effect and is likely to change the estimate. Very low quality: We are very uncertain about the estimate.

Abbreviations: BMI, body mass index; CI, confidence interval; MD, mean difference; MMN, multiple micronutrient; RR, risk ratio.

* The basis for the **assumed risk** (e.g., the median control group risk across studies) is provided in footnotes. The **corresponding risk** (and its 95% confidence interval) is based on the assumed risk in the comparison group and the **relative effect** of the intervention (and its 95% CI).

^a^ Downgraded due to very serious study limitations.

^b^ Downgraded by one level due to imprecision.

^c^ Downgraded by one level due to high heterogeneity.

**ADDITIONAL TABLES**

1. Existing systematic reviews on micronutrient interventions in adolescents

**Table:** 

Review article	Target population	Intervention reviewed	HIC or LIC	Last search date	Primary outcomes	Secondary outcomes	No. of studies	Sub group analysis	Quality assessment	Meta‐analysis MD (95% CI)
Salam Rehana et al. (2016)	Adolescents (11–19 years) and Youth (15–24 years)	Micronutrient supplementation	Global	December 2014	Outcomes were not prespecified so all the outcomes reported by the study authors were included	Outcomes were not prespecified	31	School setting	Cochrane risk of bias assessment tool	Impact of Iron‐folic acid supplementation on anaemia RR = 0.69(0.62, 0.76)
Salam Rehana et al. (2016)	Pregnant Adolescents	Micronutrient supplementation	Global	December 2014	Outcomes were not prespecified so all the outcomes reported by the study authors were included	Outcomes were not prespecified	16	Not mentioned	Cochrane risk of bias assessment tool	Impact of nutrition interventions on
		Nutritional education								Mean birth weight SMD = 0.25(0.08,0.41)
										Low birth weight RR = 0.70(0.57, 0.84)
Lassi Zohra et al. (2017)	Adolescents (10–19 years) and Women of reproductive age	Micronutrient supplementation	Global	October 2016	Mortality, pregnancy outcomes, morbidity, nutritional, anthropometrics		107	Family based interventions	GRADE Working Group grades of evidence	Iron supplementation vs. placebo:
		Food/protein energy supplementation								
		Nutrition education for pregnant adolescents								Hemoglobin concentration (g/L) in adolescents: SMD = 1.83(0.59, 3.08)
		Obesity prevention								IFA supplementation vs. placebo:
										Hemoglobin (g/L) in adolescents: MD = 2.24(0.36, 4.12)
		Management of gestational diabetes								Vitamin D supplementation vs. placebo
										25(OH)D (nmol/L) concentration in adolescents: MD = 8.80(−2.68, 20.28)
										Zinc supplementation vs. placebo:
										Hemoglobin (g/L) concentration in adolescents: SMD = 4.81(0.97, 8.66)
										Serum zinc (mol/L) in adolescents: SMD = 4.28(2.49, 6.06)
										Preterm birth in pregnant adolescents: RR = 0.57(0.46, 0.69)
										Low birth weight in pregnant adolescents: RR = 0.39(0.15, 0.98)
										Iodine supplementation vs. placebo:
										TSH (U/dL) concentration in adolescents:.SMD = 0.25(−0.02, 0.52)
										Interventions for prevention of obesity in pregnant adolescent: birth weight: SMD = −0.05(−0.11, 0.01)
										Interventions for management of obesity in adolescents: BMI:
										SMD = −0.24(−0.36, −0.13)
Hoyland, Dye and Lawton (2009)	Children or adolescent (aged 18 years)	Any type of breakfast manipulation	Global	January 2009	Outcome measures of cognitive performance		45	Not mentioned	JADAD criteria used	Not performed
Meiklejohn, Ryan and Palermo (2016)	Adolescents aged 18 years	Nutrition education was delivered in conjunction with complementary strategies	High and Middle Income Countries	September 2014	Anthropometric measures, biochemical markers, dietary consumption data, changes in dietary intake of fruits and vegetables, snack foods, fat, sucrose, sugar‐sweetened beverages and soft drinks		13	Not mentioned	American Dietetics Association. ADA	Not performed
									Evidence Analysis Manual, IV ed	
Das et al. (2013)	Children and adolescent till age of 18 years and women of reproductive age	Fortification	Global	November 2012	Serum micronutrient levels, hematologic markers, anthropometric indicators, pregnancy outcomes, morbidity outcomes, mortality		201	Age groupsCountries	GRADE Working Group grades of evidence	Results for iron fortification in children
										Hemoglobin levels: SMD = 0.55 (0.34, 0.76)
								Population characteristics		Effect on anaemia: RR = 0.55 (0.42, 0.72)
								Type of food fortified		Results for zinc fortification in children
								Duration of intervention		Serum zinc levels: SMD = 1.28 (0.56, 2.01)
										Hemoglobin level: SMD = −0.11(−0.52, 0.31)
										Copper Levels: SMD = 0.57 (−0.91, 2.06)
										Serum alkaline phosphatase levels: SMD = 0.94(−0.29, 2.17)
										Weight gain: SMD = 0.50(−0.12, 1.11)
										Height growth: SMD = 0.52 (0.01, 1.04)
										Calcium and vitamin D fortification
										Serum parathyroid hormone levels:SMD = −0.40 (−0.56,−0.24)
										Serum vitamin D levels: SMD = 1.23 (0.35,2.11)
										Serum calcium levels:SMD = −0.40 (−0.59,−0.20)
										Results for multiple micronutrient fortification in children
										Hemoglobin levels: SMD = 0.75(0.41, 1.08)
										Effect on anaemia: RR: 0.55 (0.42, 0.71)
										Effect on vitamin A deficiency: RR = 0.90 (0.76, 1.06)
										Height‐for age Z‐score: SMD: 0.13(−0.04, 0.29)
										Weight‐for age Z‐score: SMD: −0.12(−0.43, 0.20)
										Weight‐for height Zscore:SMD: −0.11(−0.40, 0.17)
										Results for iron, folate and calcium/vitamin D fortification in women
										Hemoglobin levels: SMD: 0.62 (0.36,0.89)
										Effect on anaemia: RR: 0.68 (0.49, 0.93)
Marquez, Racey, Preyde, Hendrie and Newton (2015)	Adolescents aged 12 to 18 years	Interventions targeting an increase in dairy food or Calcium intake	Global	February 2015	Intakes of calcium, milk and dairy per day		16	Not mentioned	The Quality	Not performed
									Assessment Tool for Quantitative Studies	
									by EPHPP	
Samuelson 2017	Adolescents aged 10–19 years	Diet and nutrition interventions	Global	January 2016	Depression		11	Not mentioned	Not mentioned	Not performed
Lohner et al. (2012)	Children and adolescents	Folate supplementation	Global	March 2009	Serum folate content, erythrocyte folate content		26	Not mentioned	Not mentioned	Not performed

2. WHO building blocks criteria

**Table:** 

Studies	Service delivery	Health workforce	Health information systems	Access to essential medicines/supplies	Financing	Leadership/governance
Agarwal et al. (2003)	Delivery of iron supplements in school	Probably through school teachers	Not specified	Iron supplements were provided by researchers	UNICEF, New Delhi	Researchers
Chiplonkar and Kawade (2012)	Delivery of food supplements and zinc tablets in school	Probably through school teachers	Not specified	Food supplements and zinc tablets provided by researchers	Zensar Foundation, Pune, India	Researchers
Februhartanty et al. (2002)	Delivery of iron supplements in schools	Delivered through school teachers	Not specified	Iron supplements were provided by researchers	SEAMEO‐TROPMED Regional Center for Community Nutrition in Jakarta	Researchers
Goyle 2012	Supplement biscuits in schools	Probably through school teachers	Not specified	Biscuits were supplied through researcher	University Grants Commission, New Delhi, India	Researchers
Hettiarachchi et al. (2008)	Iron and zinc supplements provided in schools	Delivered through teachers and investigators	Not specified	Supplements were provided by the researchers	The study was funded by the International Atomic Energy Agency	Researchers
Hyder et al. (2007)	Iron fortified beverage provided in school	Delivered through schoolteachers with the assistance of the BRAC community health workers	Not specified	Supplements were provided by the researchers	Supported by the Micronutrient Initiative, Ottawa, Canada	Bangladesh Rural Advancement Committee (BRAC, one of the largest national nongovernmental organizations in the world) who were the researchers
Khadilkar et al. (2010)	Vitamin D supplement were provided in school	The tablets were supplied to participants monthly by trial staff	Not specified	Supplements were provided by the researchers	Not specified	Researchers
Sen (2009)	Iron supplements were provided in schools	Investigators, monitors, class teachers	Not specified	Supplements were provided by the researchers	None	Researchers
Soekarjo et al. (2004)	Vitamin A, iron and folate supplements were provided in the schools	Field workers supervised the supplement intake	Not specified	Supplements were produced locally and provided by the researcher	This study was funded by USAID through the OMNI project	Researchers
Zhu et al. (2005)	Milk supplementation given in schools	Probably through school teachers	Not specified	Milk supplementation given in schools provided by the researchers	Australian Dairy Research and Development Corporation, Murray Goulburn Co‐operative Co. Limited (formulated and produced the milk supplements) and the Nestle' Foundation provided financial support for the laboratory analyses	Researchers

## DATA AND ANALYSES

1. Micronutrient Supplementation/Fortification versus No Supplementation/Fortificaton

**Table:** 

Outcome or subgroup	Studies	Participants	Statistical method	Effect estimate
1.1 Anaemia	1		Risk Ratio (IV, Random, 95% CI)	No totals
1.1.1 Iron Supplementation with or without Folic Acid	1		Risk Ratio (IV, Random, 95% CI)	No totals
1.2 BMI	6		Mean Difference (IV, Random, 95% CI)	Subtotals only
1.2.1 Calcium/Vitamin D Supplementation/Fortification	2	964	Mean Difference (IV, Random, 95% CI)	−0.01 [−1.20, 1.17]
1.2.2 Iron Supplementation with or without Folic Acid	2	738	Mean Difference (IV, Random, 95% CI)	0.47 [−0.17, 1.11]
1.2.3 Zinc Supplementation	1	382	Mean Difference (IV, Random, 95% CI)	0.35 [−0.15, 0.85]
1.2.4 Multiple Micronutrient Fortification	2	943	Mean Difference (IV, Random, 95% CI)	0.23 [−0.11, 0.57]
1.3 Haemoglobin	6		Mean Difference (IV, Random, 95% CI)	Subtotals only
1.3.1 Iron Supplementation with or without Folic Acid	4	1,220	Mean Difference (IV, Random, 95% CI)	0.58 [0.28, 0.88]
1.3.2 Multiple Micronutrient Fortification	2	1,102	Mean Difference (IV, Random, 95% CI)	−0.10 [−0.88, 0.68]
1.4 Micronutrient status: Serum 25(OH) D	2	517	Std. Mean Difference (IV, Random, 95% CI)	2.85 [0.89, 4.82]
1.4.1 Calcium/Vitamin D Supplementation/Fortification	2	517	Std. Mean Difference (IV, Random, 95% CI)	2.85 [0.89, 4.82]
1.5 Micronutrient status: Serum zinc levels	2	494	Std. Mean Difference (IV, Random, 95% CI)	6.94 [−4.84, 18.71]
1.5.1 Zinc Supplementation	2	494	Std. Mean Difference (IV, Random, 95% CI)	6.94 [−4.84, 18.71]
1.6 Body composition: Total body BMC	1		Mean Difference (IV, Random, 95% CI)	No totals
1.6.1 Calcium/Vitamin D Supplementation/Fortification	1		Mean Difference (IV, Random, 95% CI)	No totals
1.7 Body composition: Total body BMD	1		Mean Difference (IV, Random, 95% CI)	No totals
1.7.1 Calcium/Vitamin D Supplementation/Fortification	1		Mean Difference (IV, Random, 95% CI)	No totals
1.8 Cognitive outcomes	1		Mean Difference (IV, Random, 95% CI)	No totals
1.8.1 Digit span scores	1		Mean Difference (IV, Random, 95% CI)	No totals
1.8.2 Clerical task scores	1		Mean Difference (IV, Random, 95% CI)	No totals
1.8.3 Visual memory test scores	1		Mean Difference (IV, Random, 95% CI)	No totals
1.8.4 Maze test scores	1		Mean Difference (IV, Random, 95% CI)	No totals

## SOURCES OF SUPPORT

### Internal sources

- Aga Khan University, Pakistan.

### External sources

- Funding for this review came from a grant from the Bill & Melinda Gates Foundation to the Centre for Global Child Health at the Hospital for Sick Children, Canada.

**Feedback**

## APPENDIX A

### Search Strategy

#### **PubMed Search Strategy**: (titles/abstracts and text words)

((“Adolescent”[Mesh]) OR (“Child”[Mesh]) OR (Adolescent* OR Adolescence) OR (Teen* OR (Youth*) OR (Puberty) OR (juvenil*)) AND ((“Micronutrients”[Mesh]) OR (“Dietary Supplements”[Mesh]) OR (“Food, Fortified”[Mesh]) OR (“Vitamins”[Mesh]) OR (“Minerals”[Mesh] OR “Trace Elements”[Mesh]) OR (“Ferric compounds”[Mesh] OR “Ferrous Compounds”[Mesh]) OR (Iron* OR Ferric OR Ferrous) OR (“Diet Supplement*” OR “Dietary Supplement*” OR Biofortification) OR (“Folic Acid”[Mesh]) OR (Folic* OR Folate* OR Folvite* OR Folacin*) OR (“Zinc”[Mesh] OR “Zinc Sulfate”[Mesh]) OR (“Calcium”[Mesh]) OR (Calcium) OR (“Vitamin D”[Mesh]) OR (vitamin d) OR (“Vitamin A”[Mesh]) OR (“Vitamin A”) OR (“Ascorbic Acid”[Mesh]) OR (“Vitamin C”) OR (Ascorb* OR “ascorbic acid”) OR (Vitamin* OR multivitamin* OR multi‐vitamin* OR MMN OR micro‐nutrient* OR mineral* OR multimineral* OR multi‐mineral OR multinutrient* OR “multiple micronutrient*” OR “food environment” OR advertisement* OR “mass media” OR “supplementary feeding” OR “energy supplement*” OR “protein supplement*” OR “lipid based nutrition” OR LNS)) AND ((“Adolescent Development”[Mesh]) OR (“Adolescent Growth”) OR (“Serum Haemoglobin” OR “Serum micronutrient*” OR “Anthropometric measurement*”))

#### EBSCO CINAHL Plus

((“Adolescent”[Mesh]) OR (Adolescent* OR Adolescence) OR (Teen* OR Teenager*) OR (Youth*) OR (Puberty) OR (juvenile)) AND ((“Micronutrients”[Mesh]) OR (“Dietary Supplements”[Mesh]) OR (“Food, Fortified”[Mesh]) OR (“Vitamins”[Mesh]) OR (“Minerals”[Mesh] OR “Trace Elements”[Mesh]) OR (“Ferric compounds”[Mesh] OR “Ferrous Compounds”[Mesh]) OR (Iron* OR Ferric OR Ferrous) OR (“Diet Supplement*” OR “Dietary Supplement*” OR Biofortification) OR (“Folic Acid”[Mesh]) OR (Folic* OR Folate* OR Folvite* OR Folacin*) OR (“Zinc”[Mesh] OR “Zinc Sulfate”[Mesh]) OR (“Calcium”[Mesh]) OR (Calcium) OR (“Vitamin D”[Mesh]) OR (vitamin d) OR (“Vitamin A”[Mesh]) OR (“Vitamin A”) OR (“Ascorbic Acid”[Mesh]) OR (“Vitamin C”) OR (Ascorb* OR “ascorbic acid”) OR (Vitamin* OR multivitamin* OR multi‐vitamin* OR MMN OR micro‐nutrient* OR mineral* OR multimineral* OR multi‐mineral OR multinutrient* OR “multiple micronutrient*” OR “food environment” OR advertisement* OR “mass media” OR “supplementary feeding” OR “energy supplement*” OR “protein supplement*” OR “lipid based nutrition” OR LNS)) AND ((“Adolescent Development”[Mesh]) OR (“Adolescent Growth”) OR (“Serum Haemoglobin” OR “Serum micronutrient*” OR “Anthropometric measurement*”))

#### Cochrane Library

((“Adolescent”[Mesh]) OR (“Child”[Mesh]) OR (Adolescent* OR Adolescence) OR (Teen* OR (Youth*) OR (Puberty) OR (juvenil*)) AND ((“Micronutrients”[Mesh]) OR (“Dietary Supplements”[Mesh]) OR (“Food, Fortified”[Mesh]) OR (“Vitamins”[Mesh]) OR (“Minerals”[Mesh] OR “Trace Elements”[Mesh]) OR (“Ferric compounds”[Mesh] OR “Ferrous Compounds”[Mesh]) OR (Iron* OR Ferric OR Ferrous) OR (“Diet Supplement*” OR “Dietary Supplement*” OR Biofortification) OR (“Folic Acid”[Mesh]) OR (Folic* OR Folate* OR Folvite* OR Folacin*) OR (“Zinc”[Mesh] OR “Zinc Sulfate”[Mesh]) OR (“Calcium”[Mesh]) OR (Calcium) OR (“Vitamin D”[Mesh]) OR (vitamin d) OR (“Vitamin A”[Mesh]) OR (“Vitamin A”) OR (“”Ascorbic Acid”[Mesh]) OR (“Vitamin C”) OR (Ascorb* OR “ascorbic acid”) OR (Vitamin* OR multivitamin* OR multi‐vitamin* OR MMN OR micro‐nutrient* OR mineral* OR multimineral* OR multi‐mineral OR multinutrient* OR “multiple micronutrient*” OR “food environment” OR advertisement* OR “mass media” OR “supplementary feeding” OR “energy supplement*” OR “protein supplement*” OR “lipid based nutrition” OR LNS)) AND ((“Adolescent Development”[Mesh]) OR (“Adolescent Growth”) OR (“Serum Haemoglobin” OR “Serum micronutrient*” OR “Anthropometric measurement*”))

## References

[cl21085-bib-0001] Agarwal, K. N. , Gomber, S. , Bisht, H. , & Som, M. (2003). Anemia prophylaxis in adolescent school girls by weekly or daily iron‐folate supplementation. Indian Pediatrics, 40(4), 296–302. 12736400

[cl21085-bib-0002] Chiplonkar, S. A. , & Kawade, R. (2012). Effect of zinc‐and micronutrient‐rich food supplements on zinc and vitamin A status of adolescent girls. Nutrition, 28(5), 551–558. 22129855 10.1016/j.nut.2011.08.019

[cl21085-bib-0003] Februhartanty, J. , Dillon, D. , & Khusun, H. (2002). Will iron supplementation given during menstruation improve iron status better than weekly supplementation? Asia Pacific Journal of Clinical Nutrition, 11(1), 36–41. 11890637 10.1046/j.1440-6047.2002.00264.x

[cl21085-bib-0004] Goyle, A. , & Prakash, S. (2010). Effect of supplementation of micronutrient fortified biscuits on haemoglobin and serum iron levels of adolescent girls from Jaipur city, India. Nutrition and Food Science, 40(5), 477–484.

[cl21085-bib-0005] Goyle, A. (2012). Effect of micronutrient fortified biscuit supplementation on the weight, height and BMI of adolescent girls. Collegium Antropologicum, 36(2), 573–579. 22856247

[cl21085-bib-0006] Hettiarachchi, M. , Liyanage, C. , Wickremasinghe, R. , Hilmers, D. C. , & Abrams, S. A. (2008). The efficacy of micronutrient supplementation in reducing the prevalence of anaemia and deficiencies of zinc and iron among adolescents in Sri Lanka. European Journal of Clinical Nutrition, 62(7), 856–865. 17522609 10.1038/sj.ejcn.1602791

[cl21085-bib-0007] Hyder, S. M. , Haseen, F. , Khan, M. , Schaetzel, T. , Jalal, C. S. , Rahman, M. , … Mehansho, H. (2007). A multiple‐micronutrient‐fortified beverage affects hemoglobin, iron, and vitamin A status and growth in adolescent girls in rural Bangladesh. The Journal of Nutrition, 137(9), 2147–2153. 17709456 10.1093/jn/137.9.2147

[cl21085-bib-0008] Khadilkar, A. V. , Sayyad, M. G. , Sanwalka, N. J. , Bhandari, D. R. , Naik, S. , Khadilkar, V. V. , & Mughal, M. Z. (2010). Vitamin D supplementation and bone mass accrual in underprivileged adolescent Indian girls. Asia Pacific Journal of Clinical Nutrition, 19(4), 465–472. 21147706

[cl21085-bib-0009] Sen, A. , & Kanani, S. (2012). Intermittent iron folate supplementation: Impact on hematinic status and growth of school girls. ISRN Hematology, 2012, 482153. 22919508 10.5402/2012/482153 PMC3412096

[cl21085-bib-0010] Sen, A. , & Kanani, S. J. (2009). Physical work capacity of young underprivileged school girls: Impact of daily vs intermittent iron‐folic acid supplementation‐a randomized controlled trial. Indian Pediatrics, 46(10), 849–854. 19430082

[cl21085-bib-0011] Sen, A. , & Kanani, S. J. (2009). Impact of iron‐folic acid supplementation on cognitive abilities of school girls in Vadodara. Indian Pediatrics, 46, 2. 19242031

[cl21085-bib-0012] Soekarjo, D. D. , Pee, S. , Kusin, J. A. , Schreurs, W. H. P. , Schultink, W. , Muhilal , & Bloem, M. W. (2004). Effectiveness of weekly vitamin A (10 000 IU) and iron (60 mg) supplementation for adolescent boys and girls through schools in rural and urban East Java, Indonesia. European Journal of Clinical Nutrition, 58, 927e37–937e37. 15164114 10.1038/sj.ejcn.1601914

[cl21085-bib-0013] Xueqin, Du , Zhu, K. , Trube, A. , Zhang, Q. , Ma, G. , Hu, X. , … Greenfield, H. (2004). School‐milk intervention trial enhances growth and bone mineral accretion in Chinese girls aged 10–12 years in Beijing. British Journal of Nutrition, 92(1), 159–168. 15230999 10.1079/BJN20041118

[cl21085-bib-0014] Zhu, K. , Du, X. , Cowell, C. T. , Greenfield, H. , Blades, B. , Dobbins, T. A. , … Fraser, D. R. (2005). Effects of school milk intervention on cortical bone accretion and indicators relevant to bone metabolism in Chinese girls aged 10–12 y in Beijing. The American Journal of Clinical Nutrition, 81(5), 1168–1175. 15883444 10.1093/ajcn/81.5.1168

[cl21085-bib-0015] Zhu, K. , Zhang, Q. , Foo, L. H. , Trube, A. , Ma, G. , Hu, X. , … Greenfield, H. (2006). Growth, bone mass, and vitamin D status of Chinese adolescent girls 3 y after withdrawal of milk supplementation. The American Journal of Clinical Nutrition, 83(3), 714–721. 16522922 10.1093/ajcn.83.3.714

[cl21085-bib-0016] Abrams, S. A. , Griffin, I. J. , Hawthorne, K. M. , Gunn, S. K. , Gundberg, C. M. , & Carpenter, T. O. (2005). Relationships among vitamin D levels, parathyroid hormone, and calcium absorption in young adolescents. The Journal of Clinical Endocrinology and Metabolism, 90(10), 5576–5581. 16076940 10.1210/jc.2005-1021 PMC1283091

[cl21085-bib-0017] Ahmed, F. , Khan, M. R. , Akhtaruzzaman, M. , Karim, R. , Marks, G. C. , Banu, C. P. , … Williams, G. (2005). Efficacy of twice‐weekly multiple micronutrient supplementation for improving the hemoglobin and micronutrient status of anemic adolescent school girls in Bangladesh. The American Journal of Clinical Nutrition, 82(4), 829–835. 16210713 10.1093/ajcn/82.4.829

[cl21085-bib-0018] Ahmed, F. , Khan, M. R. , Akhtaruzzaman, M. , Karim, R. , Williams, G. , Torlesse, H. , … Nahar, B. (2010). Long‐term intermittent multiple micronutrient supplementation enhances hemoglobin and micronutrient status more than iron+ folic acid supplementation in bangladeshi rural adolescent girls with nutritional anemia. The Journal of Nutrition, 140(10), 1879–1886. 20702745 10.3945/jn.109.119123

[cl21085-bib-0019] Angeles‐Agdeppa, I. , Schultink, W. , Sastroamidjojo, S. , Gross, R. , & Karyadi, D. (1997). Weekly micronutrient supplementation to build iron stores in female Indonesian adolescents. American Journal of Clinical Nutrition, 66, 177e83–183e83. 9209187 10.1093/ajcn/66.1.177

[cl21085-bib-0020] Beasley, N. M. , Tomkins, A. M. , Hall, A. , Lorri, W. , Kihamia, C. M. , & Bundy, D. A. (2000). The impact of weekly iron supplementation on the iron status and growth of adolescent girls in Tanzania. Tropical Medicine and International Health, 5(11), 794–799. 11123827 10.1046/j.1365-3156.2000.00641.x

[cl21085-bib-0021] Castillo‐Durán, C. , Marín, V. B. , Alcázar, L. S. , Iturralde, H. , & Ruz, M. O. (2001). Controlled trial of zinc supplementation in Chilean pregnant adolescents. Nutrition Research, 21(5), 715–724.

[cl21085-bib-0022] Chan, G. M. , McElligott, K. , McNaught, T. , & Gill, G. (2006). Effects of dietary calcium intervention on adolescent mothers and newborns: A randomized controlled trial. Obstetrics and Gynecology, 108(3), 565–571. 16946216 10.1097/01.AOG.0000231721.42823.9e

[cl21085-bib-0023] Damsgaard, C. T. , Mølgaard, C. , Matthiessen, J. , Gyldenløve, S. N. , & Lauritzen, L. (2012). The effects of n‐3 long‐chain polyunsaturated fatty acids on bone formation and growth factors in adolescent boys. Pediatric Research, 71(6), 713–719. 22337227 10.1038/pr.2012.28

[cl21085-bib-0024] de Oliveira, K. D. , Donangelo, C. M. , de Oliveira, A. V., Jr , da Silveira, C. L. , & Koury, J. C. (2009). Effect of zinc supplementation on the antioxidant, copper, and iron status of physically active adolescents. Cellular Biochemistry and its Modulation by Active Agents or Disease, 27(3), 162–166. 10.1002/cbf.1550 19277992

[cl21085-bib-0025] Deshmukh, P. R. , Garg, B. S. , & Bharambe, M. S. (2008). Effectiveness of weekly supplementation of iron to control anaemia among adolescent girls of Nashik, Maharashtra, India. Journal of Health, Population, and Nutrition, 26(1), 74–78. 18637530 PMC2740684

[cl21085-bib-0026] Dibba, B. , Prentice, A. , Ceesay, M. , Stirling, D. M. , Cole, T. J. , & Poskitt, E. M. (2000). Effect of calcium supplementation on bone mineral accretion in Gambian children accustomed to a low‐calcium diet. The American Journal of Clinical Nutrition, 71(2), 544–549. 10648270 10.1093/ajcn/71.2.544

[cl21085-bib-0027] Diogenes, M. E. , Bezerra, F. F. , Rezende, E. P. , Taveira, M. F. , Pinhal, I. , & Donangelo, C. M. (2013). Effect of calcium plus vitamin D supplementation during pregnancy in Brazilian adolescent mothers: A randomized, placebo‐controlled trial. The American Journal of Clinical Nutrition, 98(1), 82–91. 23719547 10.3945/ajcn.112.056275

[cl21085-bib-0028] Diogenes, M. E. , Bezerra, F. F. , Rezende, E. P. , & Donangelo, C. M. (2015). Calcium plus vitamin D supplementation during the third trimester of pregnancy in adolescents accustomed to low calcium diets does not affect infant bone mass at early lactation in a randomised controlled trial. The Journal of Nutrition, 145(7), 1515–1523. 26019245 10.3945/jn.114.208140

[cl21085-bib-0029] Dongre, A. R. , Deshmukh, P. R. , & Garg, B. S. (2011). Community‐led initiative for control of anemia among children 6 to 35 months of age and unmarried adolescent girls in rural Wardha, India. Food and Nutrition Bulletin, 32(4), 315–323. 22590964 10.1177/156482651103200402

[cl21085-bib-0030] Eftekhari, M. H. , Simondon, K. B. , Jalali, M. , Keshavarz, S. A. , Elguero, E. , Eshraghian, M. R. , & Saadat, N. (2006). Effects of administration of iron, iodine and simultaneous iron‐plus‐iodine on the thyroid hormone profile in iron‐deficient adolescent Iranian girls. European Journal of Clinical Nutrition, 60(4), 545–552. 16340950 10.1038/sj.ejcn.1602349

[cl21085-bib-0031] Friis, H. , Ndhlovu, P. , Mduluza, T. , Kaondera, K. , Sandström, B. , Michaelsen, K. , … Christensen, N. (1997). The impact of zinc supplementation on growth and body composition: A randomized, controlled trial among rural Zimbabwean schoolchildren. European Journal of Clinical Nutrition, 51, 38–45. 9023466 10.1038/sj.ejcn.1600358

[cl21085-bib-0032] Ganmaa, D. , Stuart, J. J. , Sumberzul, N. , Ninjin, B. , Giovannucci, E. , Kleinman, K. , … Rich‐Edwards, J. W. (2017). Vitamin D supplementation and growth in urban Mongol school children: Results from two randomized clinical trials. PLOS One, 12(5):e0175237. 28481882 10.1371/journal.pone.0175237 PMC5421751

[cl21085-bib-0033] Ilich‐Ernst, J. Z. , McKenna, A. A. , Badenhop, N. E. , Clairmont, A. C. , Andon, M. B. , Nahhas, R. W. , … Matkovic, V. (1998). Iron status, menarche, and calcium supplementation in adolescent girls. The American Journal of Clinical Nutrition, 68(4), 880–887. 9771866 10.1093/ajcn/68.4.880

[cl21085-bib-0034] Kianfar, H. , Kimiagar, M. , & Ghaffarpour, M. (2000). Effect of daily and intermittent iron supplementation on iron status of high school girls. International Journal for Vitamin and Nutrition Research, 70(4), 172–177. 10989766 10.1024/0300-9831.70.4.172

[cl21085-bib-0035] Kotecha, P. V. , Nirupam, S. , & Karkar, P. D. (2009). Adolescent girls’ anaemia control programme, Gujarat, India. Indian Journal of Medical Research, 130(5), 584–589. 20090111

[cl21085-bib-0036] Lambert, H. L. , Eastell, R. , Karnik, K. , Russell, J. M. , & Barker, M. E. (2008). Calcium supplementation and bone mineral accretion in adolescent girls: An 18‐mo randomized controlled trial with 2‐y follow‐up. The American Journal of Clinical Nutrition, 87(2), 455–462. 18258639 10.1093/ajcn/87.2.455

[cl21085-bib-0037] Ma, X. M. , Huang, Z. W. , Yang, X. G. , & Su, Y. X. (2014). Calcium supplementation and bone mineral accretion in Chinese adolescents aged 12–14 years: A 12‐month, dose–response, randomised intervention trial. British Journal of Nutrition, 112(9), 1510–1520. 25231730 10.1017/S0007114514002384

[cl21085-bib-0038] Manger, M. S. , McKenzie, J. E. , Winichagoon, P. , Gray, A. , Chavasit, V. , Pongcharoen, T. , … Gibson, R. S. (2008). A micronutrient‐fortified seasoning powder reduces morbidity and improves short‐term cognitive function, but has no effect on anthropometric measures in primary school children in northeast Thailand: A randomized controlled trial. The American Journal of Clinical Nutrition, 87(6), 1715–1722. 18541560 10.1093/ajcn/87.6.1715

[cl21085-bib-0039] Mann, S. K. , Kaur, S. , & Bains, K. (2002). Iron and energy supplementation improves the physical work capacity of female college students. Food and nutrition bulletin, 23, 57e64–64e64. 10.1177/156482650202300108 11975370

[cl21085-bib-0040] McKenna, A. A. , Ilich, J. Z. , Andon, M. B. , Wang, C. , & Matkovic, V. (1997). Zinc balance in adolescent females consuming a low‐or high‐calcium diet. The American Journal of Clinical Nutrition, 65(5), 1460–1464. 9129477 10.1093/ajcn/65.5.1460

[cl21085-bib-0041] Mwaniki, D. , Omondi, B. , Muniu, E. , Thiong'o, F. , Ouma, J. , Magnussen, P. , … Friis, H. (2002). Effects on serum retinol of multi‐micronutrient supplementation and multi‐helminth chemotherapy: A randomised, controlled trial in Kenyan school children. European Journal of Clinical Nutrition, 56(7), 666–673. 12080408 10.1038/sj.ejcn.1601376

[cl21085-bib-0042] Pilz, S. , Hahn, A. , Schön, C. , Wilhelm, M. , & Obeid, R. (2017). Effect of two different multimicronutrient supplements on vitamin D status in women of childbearing age: A randomized trial. Nutrients, 9(1), 30. 28054964 10.3390/nu9010030 PMC5295074

[cl21085-bib-0043] Prentice, A. , Ginty, F. , Stear, S. J. , Jones, S. C. , Laskey, M. A. , & Cole, T. J. (2005). Calcium supplementation increases stature and bone mineral mass of 16‐to 18‐year‐old boys. The Journal of Clinical Endocrinology and Metabolism, 90(6), 3153–3161. 15755856 10.1210/jc.2004-2114

[cl21085-bib-0044] Prentice, A. , Dibba, B. , Sawo, Y. , & Cole, T. J. (2012). The effect of prepubertal calcium carbonate supplementation on the age of peak height velocity in Gambian adolescents. The American Journal of Clinical Nutrition, 96(5), 1042–1050. 22990031 10.3945/ajcn.112.037481 PMC3642996

[cl21085-bib-0045] Ward, K. A. , Cole, T. J. , Laskey, M. A. , Ceesay, M. , Mendy, M. B. , Sawo, Y. , & Prentice, A. (2014). The effect of prepubertal calcium carbonate supplementation on skeletal development in Gambian boys—A 12‐year follow‐up study. The Journal of Clinical Endocrinology & Metabolism, 99(9), 3169–3176. 24762110 10.1210/jc.2014-1150 PMC5165037

[cl21085-bib-0046] Rerksuppaphol, S. , & Rerksuppaphol, L. (2016). Effect of zinc plus multivitamin supplementation on growth in school children. Pediatrics International, 58(11), 1193–1199. 27083763 10.1111/ped.13011

[cl21085-bib-0047] Rousham, E. K. , Uzaman, B. , Abbott, D. , Lee, S. F. , Mithani, S. , Roschnik, N. , & Hall, A. (2013). The effect of a school‐based iron intervention on the haemoglobin concentration of school children in north‐west Pakistan. European Journal of Clinical Nutrition, 67, 1188e92–1192e92. 24022261 10.1038/ejcn.2013.160

[cl21085-bib-0048] Sarma, K. R. , Udaykumar, P. , Balakrishna, N. , Vijayaraghavan, K. , & Sivakumar, B. (2006). Effect of micronutrient supplementation on health and nutritional status of schoolchildren: Growth and morbidity. Nutrition, 22(1), S8–S14. 16426962 10.1016/j.nut.2005.07.011

[cl21085-bib-0049] Shatrugna, V. , Balakrishna, N. , & Krishnaswamy, K. (2006). Effect of micronutrient supplement on health and nutritional status of schoolchildren: Bone health and body composition. Nutrition, 22(1), S33–S39. 16426961 10.1016/j.nut.2005.07.010

[cl21085-bib-0050] Vazir, S. , Nagalla, B. , Thangiah, V. , Kamasamudram, V. , & Bhattiprolu, S. (2006). Effect of micronutrient supplement on health and nutritional status of schoolchildren: Mental function. Nutrition, 22(1), S26–S32. 16426960 10.1016/j.nut.2004.07.021

[cl21085-bib-0051] Schou, A. J. , Heuck, C. , & Wolthers, O. D. (2003). A randomized, controlled lower leg growth study of vitamin D supplementation to healthy children during the winter season. Annals of Human Biology, 30(2), 214–219. 12637196 10.1080/0301446021000057629

[cl21085-bib-0052] Shah, B. K. , & Gupta, P. (2002). Weekly vs daily iron and folic acid supplementation in adolescent Nepalese girls. Archives of Pediatrics and Adolescent Medicine, 156(2), 131–135. 11814373 10.1001/archpedi.156.2.131

[cl21085-bib-0053] Silk, L. N. , Greene, D. A. , Baker, M. K. , & Jander, C. B. (2015). Tibial bone responses to 6‐month calcium and vitamin D supplementation in young male jockeys: A randomised controlled trial. Bone, 81, 554–561. 26362226 10.1016/j.bone.2015.09.004

[cl21085-bib-0054] Sunawang, U. B. , Hidayat, A. , & Kusharisupeni, S. (2009). Preventing low birthweight through maternal multiple micronutrient supplementation: A cluster‐randomized, controlled trial in Indramayu, West Java. Food and Nutrition Bulletin, 4(4), S488–S495. 10.1177/15648265090304S403 20120790

[cl21085-bib-0055] Tee, E. S. , Kandiah, M. , Awin, N. , Chong, S. M. , Satgunasingam, N. , Kamarudin, L. , … Viteri, F. E. (1999). School‐administered weekly iron‐folate supplements improve hemoglobin and ferritin concentrations in Malaysian adolescent girls. American Journal of Clinical Nutrition, 69, 1249e56–1256e56. 10357747 10.1093/ajcn/69.6.1249

[cl21085-bib-0056] Viljakainen, H. T. , Natri, A. M. , Kärkkäinen, M. , Huttunen, M. M. , Palssa, A. , Jakobsen, J. , … Lamberg‐Allardt, C. (2006). A Positive Dose–Response Effect of Vitamin D supplementation on site‐specific bone mineral augmentation in adolescent girls: A double‐blinded randomized placebo‐controlled 1‐year intervention. Journal of Bone and Mineral Research, 21(6), 836–844. 16753014 10.1359/jbmr.060302

[cl21085-bib-0057] White, D. J. , Cox, K. H. , Peters, R. , Pipingas, A. , & Scholey, A. B. (2015). Effects of four‐week supplementation with a multi‐vitamin/mineral preparation on mood and blood biomarkers in young adults: A randomised, double‐blind, placebo‐controlled trial. Nutrients, 7(11), 9005–9017. 26529011 10.3390/nu7115451 PMC4663579

[cl21085-bib-0058] Yusoff, H. , Wan Daud, W. N. , & Ahmad, Z. (2012). Nutrition education and knowledge, attitude and hemoglobin status of Malaysian adolescents. Southeast Asian Journal of Tropical Medicineand Public Health, 43(1), 192–200. 23082570

[cl21085-bib-0059] Akseer, N. , Al‐Gashm, S. , Mehta, S. , Mokdad, A. , & Bhutta, Z. A. (2017). Global and regional trends in the nutritional status of young people: A critical and neglected age group. Annals of the New York Academy of Sciences, 1393(1), 3–20. 28436100 10.1111/nyas.13336

[cl21085-bib-0060] Allen, L. H. , De Benoist, B. , Dary, O. , & Hurrell, R. , World Health Organization . (2006). Guidelines on food fortification with micronutrients. World Health Organization.

[cl21085-bib-0061] Ameratunga, S. N. (2017). Country‐level data informing a sustainable development agenda for adolescents. Journal of Adolescent Health, 61(4), 405–406. 10.1016/j.jadohealth.2017.07.014 28941482

[cl21085-bib-0062] Baldasso, J. G. , Galante Andrea, P. , & De Piano Ganen, A. (2016). Impact of actions of food and nutrition education program in a population of adolescents. Revista de Nutrição, 29(1), 65–75.

[cl21085-bib-0063] Balshem, H. , Helfand, M. , Schünemann, H. J. , Oxman, A. D. , Kunz, R. , … Brozek, J. (2011). GRADE guidelines: 3. Rating the quality of evidence. Journal of Clinical Epidemiology, 64(4), 401–406. 21208779 10.1016/j.jclinepi.2010.07.015

[cl21085-bib-0064] Blasbalg Tanya, L. , Wispelwey, B. , & Deckelbaum Richard, J. (2011). Econutrition and utilization of food‐based approaches for nutritional health. Food and Nutrition Bulletin, 32(1_suppl1), S4–S13. 21717913 10.1177/15648265110321S102

[cl21085-bib-0065] Caleyachetty, R. , Thomas, G. N. , Kengne, A. P. , Echouffo‐Tcheugui, J. B. , Schilsky, S. , Khodabocus, J. , & Uauy, R. (2018). The double burden of malnutrition among adolescents: Analysis of data from the Global School‐Based Student Health and Health Behavior in School‐Aged Children surveys in 57 low‐and middle‐income countries. The American Journal of Clinical Nutrition, 108(2), 414–424. 29947727 10.1093/ajcn/nqy105

[cl21085-bib-0066] Contento, I. , Balch George, I. , Bronner Yvonne, L. , Lytle, L. A. , Maloney, S. K. , Olson, C. M. , & Swadener, SS (1995). The effectiveness of nutrition education and implications for nutrition education policy, programs, and research: A review of research. Journal of Nutrition Education, 27(6), 284–418.

[cl21085-bib-0067] Das, J. K. , Salam, R. A. , Kumar, R. , & Bhutta, Z. A. (2013). Micronutrient fortification of food and its impact on woman and child health: A systematic review. Systematic Reviews, 2(1), 67. 23971426 10.1186/2046-4053-2-67 PMC3765883

[cl21085-bib-0068] Dewey Kathryn, G. , & Arimond, M. (2012). Lipid‐based nutrient supplements: How can they combat child malnutrition? PLOS Medicine, 9(9):e1001314. 23028264 10.1371/journal.pmed.1001314 PMC3445442

[cl21085-bib-0069] Cochrane Effective Practice and Organisation of Care (EPOC) . Suggested risk of bias criteria for EPOC reviews . EPOC Resources for review authors. Retrieved from http://epoc.cochrane.org/resources/epoc‐resources‐review‐authors 2017.

[cl21085-bib-0070] Forouzanfar, M. H. , Alexander, L. , Anderson, H. R. , Bachman, V. F. , Biryukov, S. , … Brauer, M. (2015). Global, regional, and national comparative risk assessment of 79 behavioural, environmental and occupational, and metabolic risks or clusters of risks in 188 countries, 1990‐2013: A systematic analysis for the Global Burden of Disease Study 2013. The Lancet, 386(10010), 2287–2323. 10.1016/S0140-6736(15)00128-2 PMC4685753 26364544

[cl21085-bib-0071] Haider, B. A. , & Bhutta, Z. A. (2017). Multiple‐micronutrient supplementation for women during pregnancy. Cochrane Database of Systematic Reviews, 4, CD004905. 10.1002/14651858.CD004905.pub5 28407219 PMC6478115

[cl21085-bib-0072] Harrison, G. G. (2010). Public health interventions to combat micronutrient deficiencies. Public Health Reviews, 32(1), 256–266.

[cl21085-bib-0073] Hess Sonja, Y. , Lönnerdal, Bo , Christine, Hotz , Rivera Juan, A. , & Brown Kenneth, H. (2009). Recent advances in knowledge of zinc nutrition and human health. Food and Nutrition Bulletin, 30(1_suppl1), S5–S11. 19472599 10.1177/15648265090301S102

[cl21085-bib-0074] Higgins, J. P. , & Green, S. (2011). Cochrane handbook for systematic reviews of interventions (4). Hoboken, NJ: John Wiley & Sons.

[cl21085-bib-0075] Higgins, J P T , Altman, D G , & Sterne, J A C E (2011a). Chapter 8: Assessing risk of bias in included studies. In J. P. T. Higgins & S. Green (Eds.), Cochrane Handbook for Systematic Reviews of Interventions Version 5.1.0. Chichester, UK: Wiley‐Blackwell.

[cl21085-bib-0076] Hoyland, A. , Dye, L. , & Lawton, C. L. (2009). A systematic review of the effect of breakfast on the cognitive performance of children and adolescents. Nutrition Research Reviews, 22(2), 220–243. 19930787 10.1017/S0954422409990175

[cl21085-bib-0077] IGME . (2017). Levels & trends in child mortality: Report. Estimates developed by the UN Inter‐agency Group for Child Mortality Estimation

[cl21085-bib-0078] Jayachandran, S. (2015). The roots of gender inequality in developing countries. Economics, 7(1), 63–88.

[cl21085-bib-0079] Kroeze, W. , Werkman, A. , & Brug, J. (2006). A systematic review of randomized trials on the effectiveness of computer‐tailored education on physical activity and dietary behaviors. Annals of Behavioral Medicine, 31(3), 205–223. 16700634 10.1207/s15324796abm3103_2

[cl21085-bib-0080] Ladipo Oladapo, A. (2000). Nutrition in pregnancy: Mineral and vitamin supplements‐. The American Journal of Clinical Nutrition, 72(1), 280S–290SS. 10871594 10.1093/ajcn/72.1.280S

[cl21085-bib-0081] Lassi Zohra, S. , Anoosh, M. , Das Jai, K. , Salam Rehana, A. , & Bhutta Zulfiqar, A. (2017). Systematic review on evidence‐based adolescent nutrition interventions. Annals of the New York Academy of Sciences, 1393(1), 34–50. 28436101 10.1111/nyas.13335

[cl21085-bib-0082] Lohner, S. , Fekete, K. , Berti, C. , Hermoso, M. , Cetin, I. , Koletzko, B. , & Decsi, T. (2012). Effect of folate supplementation on folate status and health outcomes in infants, children and adolescents: A systematic review. International Journal of Food Sciences and Nutrition, 63(8), 1014–1020. 22574624 10.3109/09637486.2012.683779

[cl21085-bib-0083] Marquez, O. , Racey, M. , Preyde, M. , Hendrie, G. A. , & Newton, G. (2015). Interventions to increase dairy consumption in adolescents: A systematic review. Infant, Child, and Adolescent Nutrition, 7(5), 242–254.

[cl21085-bib-0084] Meiklejohn, S. , Ryan, L. , & Palermo, C. (2016). A systematic review of the impact of multi‐strategy nutrition education programs on health and nutrition of adolescents. Journal of Nutrition Education and Behavior, 48(9), 631–646. 27720105 10.1016/j.jneb.2016.07.015

[cl21085-bib-0085] Mengistu, K. , Alemu, K. , & Destaw, B. (2013). Prevalence of malnutrition and associated factors among children aged 6‐59 months at Hidabu Abote District, North Shewa, Oromia Regional State. Journal of Nutritional Disorders and Therapy, 1, 1–15.

[cl21085-bib-0086] Oenema, A. , Brug, J. , & Lechner, L. (2001). Web‐based tailored nutrition education: Results of a randomized controlled trial. Health Education Research, 16(6), 647–660. 11780705 10.1093/her/16.6.647

[cl21085-bib-0087] Onyango, A. W. (2013). Promoting healthy growth and preventing childhood stunting: A global challenge. Maternal and child Nutrition, 9(S2), 1–5. 10.1111/mcn.12092 PMC6860622 24074314

[cl21085-bib-0088] Patton, G. C. , Sawyer, S. M. , Santelli, J. S. , Ross, D. A. , Afifi, R. , Allen, N. B. , … Viner, R. M. (2016). Our future: A Lancet commission on adolescent health and wellbeing. The Lancet, 387(10036), 2423–2478. 10.1016/S0140-6736(16)00579-1 PMC5832967 27174304

[cl21085-bib-0089] Peyrin‐Biroulet, L. , Williet, N. , & Cacoub, P. (2015). Guidelines on the diagnosis and treatment of iron deficiency across indications: A systematic review. The American Journal of Clinical Nutrition, 102(6), 1585–1594. 26561626 10.3945/ajcn.114.103366

[cl21085-bib-0090] Pérez‐Rodrigo, C. , & Aranceta, J. (2001). School‐based nutrition education: Lessons learned and new perspectives. Public Health Nutrition, 4(1a), 131–139. 11255503 10.1079/phn2000108

[cl21085-bib-0091] Reid Ian, R. (2014). Should we prescribe calcium supplements for osteoporosis prevention? Journal of Bone Metabolism, 21(1), 21–28. 24707464 10.11005/jbm.2014.21.1.21 PMC3970298

[cl21085-bib-0092] Revman . Review Manager (RevMan) [Computer program]. Copenhagen: The Nordic Cochrane Centre, The Cochrane Collaboration 2014.

[cl21085-bib-0093] Riebl, S. K. , Estabrooks, P. A. , Dunsmore, J. C. , Savla, J. , Frisard, M. I. , Dietrich, A. M. , … Davy, B. M. (2015). A systematic literature review and meta‐analysis: The theory of planned behavior's application to understand and predict nutrition‐related behaviors in youth. Eating behaviors, 18, 160–178. 26112228 10.1016/j.eatbeh.2015.05.016

[cl21085-bib-0094] Salam, R. A. , Hooda, M. , Das, J. K. , Arshad, A. , Lassi, Z. S. , Middleton, P. , & Bhutta, Z. A. (2016). Interventions to improve adolescent nutrition: A systematic review and meta‐analysis. Journal of Adolescent Health, 59(4), S29–S39. 10.1016/j.jadohealth.2016.06.022 PMC5026685 27664593

[cl21085-bib-0095] Samuelson, R. (2017). The impact of diet and nutrition on adolescent depression: A systematic review. Master of Social Work Clinical Research Papers, 786.

[cl21085-bib-0096] Sawyer, S. M. , Afifi, R. A. , Bearinger, L. H. , Blakemore, S. J. , Dick, B. , Ezeh, A. C. , & Patton, G. C. (2012). Adolescence: A foundation for future health. The Lancet, 379(9826), 1630–1640. 10.1016/S0140-6736(12)60072-5 22538178

[cl21085-bib-0097] Sguassero, Y. , de Onis, M. , Bonotti Ana, M. , & Carroli, G. (2012). Community‐based supplementary feeding for promoting the growth of children under five years of age in low and middle income countries. The Cochrane Library, 6, CD005039. 10.1002/14651858.CD005039.pub3 PMC8078353 22696347

[cl21085-bib-0098] Stang Jamie, S. , & Stotmeister, B. (2017). Nutrition in adolescence. In N. J. Temple , T. Wilson & G. A. Bray (Eds.), Nutrition guide for physicians and related healthcare professionals (pp. 29–39). Cham, Switzerland: Springer.

[cl21085-bib-0099] Stanger, O. , Fowler, B. , Piertzik, K. , Huemer, M. , Haschke‐Becher, E. , Semmler, A. , … Linnebank, M. (2009). Homocysteine, folate and vitamin B12 in neuropsychiatric diseases: Review and treatment recommendations. Expert Review of Neurotherapeutics, 9(9), 1393–1412. 19769453 10.1586/ern.09.75

[cl21085-bib-0100] Story, M. , Lytle Leslie, A. , Birnbaum Amanda, S. , & Perry Cheryl, L. (2002). Peer‐led, school‐based nutrition education for young adolescents: Feasibility and process evaluation of the teens study. Journal of School Health, 72(3), 121–127. 11962228 10.1111/j.1746-1561.2002.tb06529.x

[cl21085-bib-0101] UNICEF . (2005). Childhood under threat: The state of the world's children 2005. UNICEF.

[cl21085-bib-0102] UNPFA . (2014). The Power of 1.8 Billion: Adolescents, Youth and the transformation of the future. UNPFA.

[cl21085-bib-0103] Walker, N. , Fischer‐Walker, C. , Bryce, J. , Bahl, R. , & Cousens, S. (2010). Effects writing for the CHERG Review Groups on Intervention. Standards for CHERG reviews of intervention effects on child survival. International Journal of Epidemiology, 39(suppl_1), i21–i31. 20348122 10.1093/ije/dyq036 PMC2845875

[cl21085-bib-0104] WHO . (2002). The World Health Report 2002—Reducing risks, promoting healthy life. Geneva: World Health Organization.

[cl21085-bib-0105] WHO (2010). Monitoring the building blocks of health systems: A handbook of indicators and their measurement strategies. Geneva: World Health Organization.

[cl21085-bib-0106] WHO (2014). Health for the world's adolescents: A second chance in the second decade: Summary. Geneva: World Health Organisation.

[cl21085-bib-0107] World Bank Country and Lending Groups‐Country Classification . The World Bank . Retrieved from https://datahelpdesk.worldbank.org/knowledgebase/articles/906519‐world‐bank‐country‐and‐lending‐groups

[cl21085-bib-0108] Zimmermann Michael, B. , & Boelaert, K. (2015). Iodine deficiency and thyroid disorders. The Lancet Diabetes and Endocrinology, 3(4), 286–295. 25591468 10.1016/S2213-8587(14)70225-6

[cl21085-bib-0109] Zimmermann, M. B. H. , & Richard, F. (2007). Nutritional iron deficiency. The Lancet, 370(9586), 511–520. 10.1016/S0140-6736(07)61235-5 17693180

[cl21085-bib-0110] Zlotkin Stanley, H. , Schauer, C. , Christofides, A. , Sharieff, W. , Tondeur Mélody, C. , & Ziauddin, H. S. M. (2005). Micronutrient sprinkles to control childhood anaemia. PLOS Medicine, 2(1), e1. 15696200 10.1371/journal.pmed.0020001 PMC545194

